# Hypervalent Iodine Reagents in High Valent Transition Metal Chemistry

**DOI:** 10.3390/molecules22050780

**Published:** 2017-05-12

**Authors:** Felipe Cesar Sousa e Silva, Anthony F. Tierno, Sarah E. Wengryniuk

**Affiliations:** Department of Chemistry, Temple University, 1901 N. 13th St., Philadelphia, PA 19122, USA; tug08706@temple.edu (F.C.S.S.); tuh08431@temple.edu (A.F.T.)

**Keywords:** hypervalent iodine, oxidation, oxidant, redox, high valent, high oxidation state, catalysis

## Abstract

Over the last 20 years, high valent metal complexes have evolved from mere curiosities to being at the forefront of modern catalytic method development. This approach has enabled transformations complimentary to those possible via traditional manifolds, most prominently carbon-heteroatom bond formation. Key to the advancement of this chemistry has been the identification of oxidants that are capable of accessing these high oxidation state complexes. The oxidant has to be both powerful enough to achieve the desired oxidation as well as provide heteroatom ligands for transfer to the metal center; these heteroatoms are often subsequently transferred to the substrate via reductive elimination. Herein we will review the central role that hypervalent iodine reagents have played in this aspect, providing an ideal balance of versatile reactivity, heteroatom ligands, and mild reaction conditions. Furthermore, these reagents are environmentally benign, non-toxic, and relatively inexpensive compared to other inorganic oxidants. We will cover advancements in both catalysis and high valent complex isolation with a key focus on the subtle effects that oxidant choice can have on reaction outcome, as well as limitations of current reagents.

## 1. Introduction

**Figure 1:**
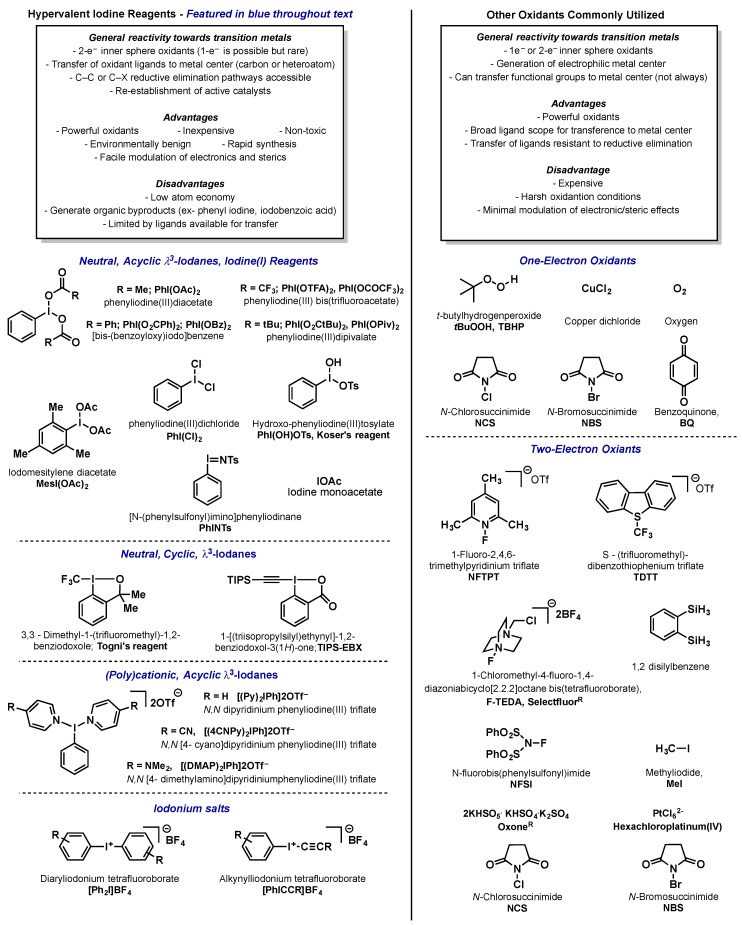
Common oxidants utilized in high valent metal catalysis.

Over the last 20 years, high valent metal complexes have transitioned from mere curiosities to being at the forefront of modern catalytic method development. This approach has enabled transformations complimentary to those possible via traditional manifolds, most prominently carbon-heteroatom bond formation. Key to the advancement of this chemistry has been the identification of oxidants that are capable of accessing these high oxidation state complexes. The oxidant has to be both powerful enough to achieve the desired oxidation as well as provide heteroatom ligands for transfer to the metal center; these heteroatoms are often subsequently transferred to the substrate via reductive elimination. The choice of heteroatom can be critical depending on the application. For example, chloride ligands can aide in the stabilization and isolation of high valent complexes whereas acetate ligands are often more successful in catalytic manifolds. Hypervalent iodine reagents have seen wide application in this field as they are environmentally benign, non-toxic, and relatively inexpensive compared to other inorganic oxidants. Furthermore, they provide an excellent balance of versatile reactivity, heteroatom ligands, and mild reaction conditions. We will cover advancements in the use of hypervalent iodine reagents for both catalysis and high valent complex isolation with a key focus on the subtle effects that oxidant choice can have on reaction outcome, as well as limitations of current reagents. Many of these areas have been covered in the context of more broad reviews and in those cases the discussion will not be comprehensive but focus on the key aspects and most relevant elements for this review. The discussion is organized broadly by the metal center being oxidized, including palladium, platinum, gold, nickel, copper, and finally isolated examples with other transition metals. Below a summary graphic has been provided that includes oxidants common to high valent transition metal chemistry that will be discussed in this review as well as common nomenclature and how they will be presented in the text (Figure 1).

## 2. Palladium

### 2.1. Introduction

**Scheme 1:**
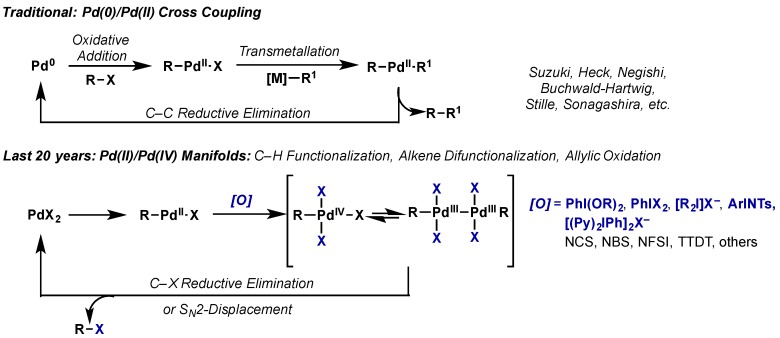
Manifolds for palladium catalysis. Traditional methods via Pd(0)/Pd(II) and recent advances in Pd(II)/Pd(IV) catalysis.

Palladium has a long and storied history in transition metal catalysis, facilitating such iconic cross coupling reactions as the Heck, Suzuki-Miyara, Negishi, Buchwald-Hartwig, Sonagashira, and others. Its ubiquity stems not only from its excellent reactivity, but also from a detailed understanding of its underlying mechanisms and predictable reactivity, which facilitates novel reaction development. For many years, palladium catalysis relied on the Pd(0)/Pd(II) redox couple, however, work in the 21st century has shown the power and promise of the high oxidation state Pd(II)/Pd(IV) manifold, as well as the potential role of Pd(III) species in catalysis (Scheme 1). This chemistry has enabled transformations previously inaccessible via traditional catalytic manifolds, most notably carbon-heteroatom bond forming reductive eliminations, the microscopic reverse of the oxidative addition pathways commonly encountered in low valent palladium catalysis. In this context, hypervalent iodine reagents have emerged as key players, facilitating net two-electron oxidations at the metal center accompanied by transfer of their heteroatom ligands, most commonly acetate or chloride. Arguably the rapid advancements and synthetic applications of high valent palladium chemistry have spurred investigations into more obscure oxidation states with other metals. As both synthetic applications and mechanistic details of this area have been comprehensively discussed in several excellent reviews in recent years [1,2,3,4,5,6,7,8], this section will discuss the key role that hypervalent iodine reagents have played in its development, with special attention paid to recent reports as well as limitations of current methods that could be addressed through continued exploration of novel oxidants. As the body of work in this area is extensive, this section will be organized based on the type of bond formation being targeted, namely C–O, C–X, C–N, and C–C bonds, and finally studies focusing on Pd(III) species.

### 2.2. Palladium(IV)

#### 2.2.1. Introduction

**Scheme 2:**
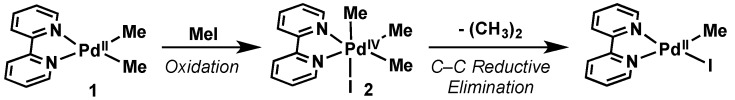
Canty’s report resulting in the first X-ray crystallographic characterization of a Pd(IV) alkyl species.

Canty reported the first X-ray structure of an alkyl Pd(IV) organometallic complex in 1986, formed via the oxidative addition of iodomethane to a dimethyl (bpy)Pd(II) complex (**1**, Scheme 2) [9]. Canty’s work revealed the octahedral geometry of Pd(IV) species **1**, as well as the clean oxidative addition/reductive elimination reactivity and stability of these complexes, which he comments could “suggest that development of a rich organometallic chemistry of palladium(IV) may be possible”. This report laid the foundation for the rich chemistry, mechanistic and structural understanding of this high oxidation state redox couple that has emerged over the last 20 years.

Pd(II)/Pd(IV) chemistry has become as a powerful synthetic manifold to facilitate transformations not accessible via traditional Pd(0)/Pd(II) catalysis (Scheme 1). This field has been pioneered by the Sanford group in the area of C–H acetoxylation and halogenation. There have been several comprehensive reviews written on this topic in recent years and the reader is directed there for detailed mechanistic discussion and an exhaustive report of applications [1,4,5]. Building from this work, Pd(IV) chemistry has been applied to the formation of a diverse array of C–N, C–O, C–X, and C–C bond forming reactions.

#### 2.2.2. Carbon–Oxygen Bond Formation

There has been extensive work in the area of C–O bond formation via Pd(II)/Pd(IV) catalysis with hypervalent iodine reagents. This application is particularly well suited as the most common hypervalent iodine oxidants, of the type PhI(OR)_2_, transfer carboxylate ligands to the metal center that are then engaged in subsequent C–O bond forming reductive elimination. These carboxylate ligands are also highly tunable, both sterically and electronically, allowing for control of complex stability, reaction pathway and selectivity. Approaches have expanded to include C–H functionalization, allylic oxidation, as well as alkene difunctionalization.

##### 2.2.2.1. C–H Functionalization

**Scheme 3:**
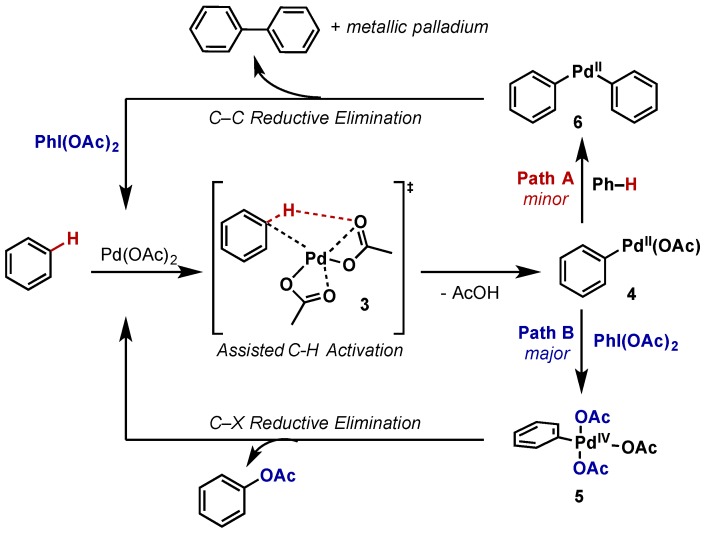
Pd(II)/Pd(IV) catalyzed acetoxylation of benzene with use of PhI(OAc)_2_ as external oxidant.

The first report of Pd-catalyzed C–H acetoxylation using a hypervalent iodine oxidant was by Crabtree, who achieved the acetoxylation of benzene using Pd(OAc)_2_ with PhI(OAc)_2_ as the external oxidant and –OAc source (Scheme 3) [10]. This built upon the work of Stock et al., which employed dichromate to perform an analogous transformation [11], however PhI(OAc)_2_ offered much higher selectivity and did not perform further product oxidations. In what has become a standard mechanistic proposal, Crabtree proposed acetyl assisted C–H activation at Pd(II) (intermediate **3**) would give intermediate **4**, which can then be diverted down one of two pathways. Along the desired pathway (Path B) **5** is then oxidized by PhI(OAc)_2_ to Pd(IV) species **5** with introduction of two acetyl groups. Subsequent reductive elimination gives rise to acetoxylated product and regeneration of the Pd(OAc)_2_. Importantly, Crabtree noted that competitive formation of biphenyl (via Path A) is minimized with PhI(OAc)_2_, indicating that oxidation to Pd(IV) in this system is significantly faster than a second C–H activation step to give **6**. It was also found that in the absence of oxidant, biphenyl was the only observable product, thus indicating that Pd(II) intermediate **4** will not undergo direct C–X reductive elimination, and emphasizing the significance of Pd(IV) pathways in facilitating such transformations. While this system displayed only moderate catalytic activity and required solvent quantities of the arene, it laid the foundation for the development of directed C–H acetoxylation, which has relied on hypervalent iodine oxidants to efficiently acetoxylate C(sp^2^)–H as well as C(sp^3^)–H bonds.

**Scheme 4:**
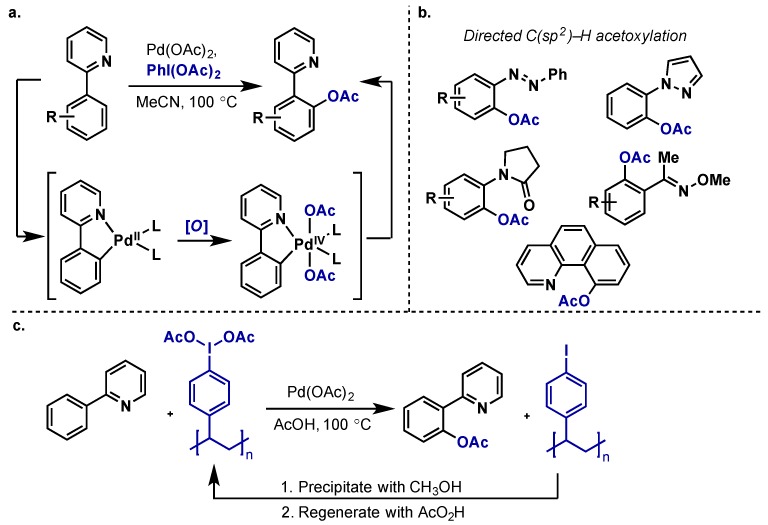
(**a**) First report on the directed C(sp^2^)–H acetoxylation of 2-phenylpyridine with Pd(OAc)_2_/PhI(OAc)_2_; (**b**) General scope of directing groups used for C(sp^2^)–H acetoxylation; (**c**) Polymer-supported λ^3^-iodane allows for facile oxidant recycling.

The Sanford group’s contributions to this area began in 2004 with their seminal report on the directed C–H acetoxylation of 2-phenylpyridine using Pd(OAc)_2_/PhI(OAc)_2_ (Scheme 4a) [12]. Since then, they have extended this method to include a range of directing groups for C(sp^2^)–H acetoxylation and a brief overview is included in Scheme 4b [13]. While other oxidants including Mn(OAc)_2_ and Oxone have been used in this chemistry, PhI(OAc)_2_ is by far the most common [2]. Sanford also demonstrated a polymer supported variant of this chemistry, which allows for facile recycling of the hypervalent iodine reagent, addressing the issue of iodobenzene byproducts produced in these transformations (Scheme 4c) [14].

**Scheme 5:**
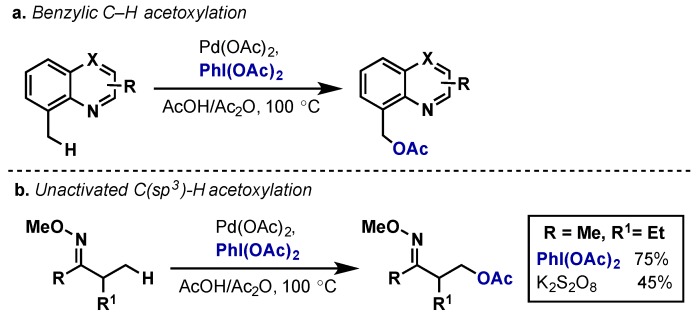
(**a**) Directed acetoxylation of benzylic C(sp^3^)–H bonds; (**b**) Directed acetoxylation of unactivated C(sp^3^)–H bonds.

The acetoxylation of both benzylic and unactivated C(sp^3^)–H bonds can also be accomplished with Pd(OAc)_2_ and PhI(OAc)_2_ (Scheme 5) [12,15,16]. In these reactions, sterics plays a large role in dictating regioselectivity, with the less sterically hindered C–H bond undergoing C–H activation. While the C(sp^3^)–H variant often performs most efficiently with PhI(OAc)_2_ as the oxidant, combinations of Oxone/Mn(OAc)_2_, molecular oxygen, and peroxide oxidants have also been used effectively [2]. Another interesting example used iodine(I) reagent IOAc, which was generated in situ from PhI(OAc)_2_ and I_2_; PhI(OAc)_2_ alone was ineffective in this case [17].

**Scheme 6:**
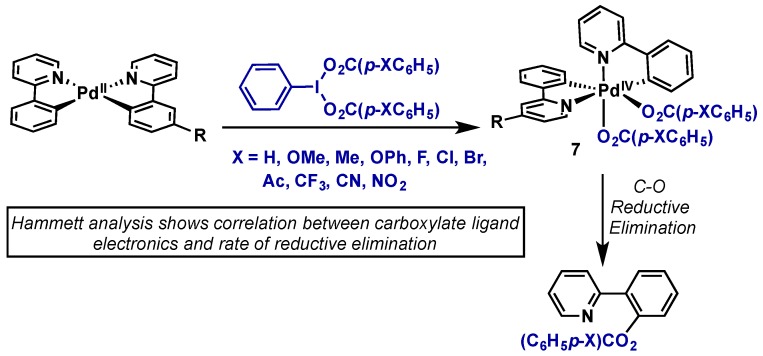
Seminal mechanistic investigation into Pd(IV)-mediated C–H acetoxylation.

The Sanford group has conducted extensive mechanistic studies on these processes, and the reader is directed to some selected full reports for detailed discussion [2,18,19,20]. Their work has been accompanied by that of Ritter and others, with a key point being whether Pd(IV) species or Pd(III) dimers are the active catalysts. Key reports detailing the possibility of Pd(III) intermediates are discussed in more detail in Section 2.3. Sanford’s seminal report in this area outlines some of the key features of both the ligand scaffolds and hypervalent iodine oxidants that allow for study of reactive Pd(IV) intermediates as well as mechanistic elucidation (Scheme 6) [19]. Two rigid cyclometallated 2-phenylpyridine ligands were incorporated to lend stability to the resultant complexes, and suppress competing ligand exchange and side reactions upon oxidation. Additionally, the acetate ligands of PhI(OAc)_2_ were exchanged for aryl carboxylates that could be readily derivatized and thus used as facile handles to control the electronic parameters at the metal center. The Pd(IV) complex (**7**) obtained upon oxidation with PhI(CO_2_*p*–XAr)_2_ where X = NO_2_ was able to characterized by X-ray crystallography, revealing *cis* addition of the two carboxylates ligands. Subsequent Hammett analysis revealed a clear correlation between carboxylate electronics and the rate of reductive elimination, indicating that the carboxylate acted in as a nucleophile in reductive elimination.

**Scheme 7:**
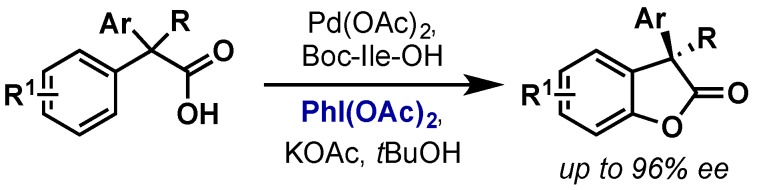
Enantioselective intramolecular C(sp^2^)–H acetoxylation using chiral amino acid ligand.

The Yu group reported an intramolecular C(sp^2^)–H acetoxylation that could also be rendered asymmetric using a Boc-Ile-OH chiral ligand (Scheme 7) [21]. This report was the first enantioselective application of Pd(II)/Pd(IV) catalysis and gave high yields and enantioselectivities of benzofuranones.

**Scheme 8:**
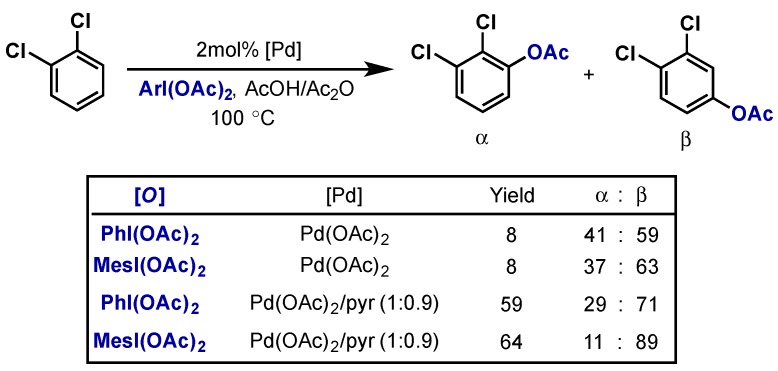
Non-directed arene C–H acetoxylation. Effect of both catalyst and hypervalent iodine oxidant on reactivity.

Sanford demonstrated an impressive example of non-directed C(sp^2^)–H acetoxylation through the addition of pyridine to enhance catalytic activity of the palladium catalyst (Scheme 8) [22]. In this system, the ratio of [Pd]/pyridine proved critical as well as the selection of hypervalent iodine oxidant. Switching to the more sterically hindered MesI(OAc)_2_ from PhI(OAc)_2_ improved both yield and regioselectivity.

**Scheme 9:**
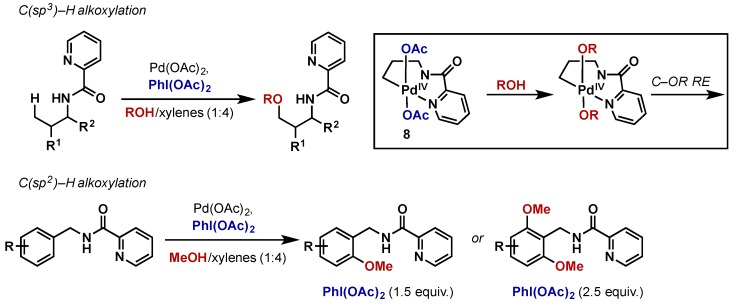
C(sp^3^)–H alkoxylation using picolinamide directing group via ligand exchange at Pd(IV).

Chen reported C(sp^3^)–H and C(sp^2^)–H alkoxylation using a picolinamide directing group (Scheme 9) [23]. The reaction is proposed to proceed via displacement of acetate ligands by alkoxides at a Pd(IV) intermediate (**8**). The authors rule out an alternative S_N_2-displacement of Pd(IV) by ROH since *t*-BuOH also participates to give C–OR bond formation. This reactivity is divergent from their previous reports on C–H amination using this same system, where **8** would undergo selective C–N reductive elimination in the absence of an external nucleophile (for discussion of C–N bond formation, see Section 2.2.4.2, Scheme 27b). It was found that the use of other oxidants including AgOAc, Oxone, Ce(SO_4_)_2_, K_2_S_2_O_8_, “F^+^” sources, as well as hypervalent iodine oxidants with other carboxylate ligands all gave inferior conversions. In C(sp^2^)–H alkoxylation, either mono- or bisalkoxylation could be achieved by altering the equivalents of PhI(OAc)_2_.

**Scheme 10:**
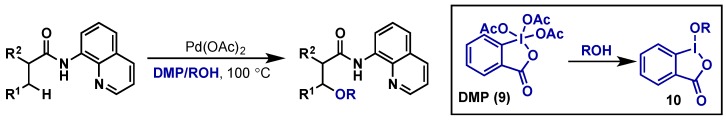
C(sp^3^)–H alkoxylation of methylene positions using an 8-aminoquinoline directing group and DMP as an oxidant.

A similar transformation has also been reported by Rao using an 8-aminoquinoline directing group and the unique choice of λ^5^-iodane Dess-Martin Periodinane (DMP, **9**) as the oxidant (Scheme 10) [24]. Other oxidants including PhI(OAc)_2_, PhI(OTFA)_2_, K_2_S_2_O_8_, NaIO_4_, NaIO_3_, and Selectfluor all gave little or no conversion to desired products and competing functionalization of the 8-aminoquinoline directing group was also observed. The authors propose that DMP is not the terminal oxidant but rather cyclic λ^3^-iodane **10**, formed in situ by attack of the alcohol on DMP, which then transfers the alkoxide to the palladium center upon oxidation. However, a similar ligand displacement at a Pd(IV) intermediate, analogous to Chen’s report, cannot be ruled out.

##### 2.2.2.2. Alkene Difunctionalization, Allylic Oxidation

**Scheme 11:**
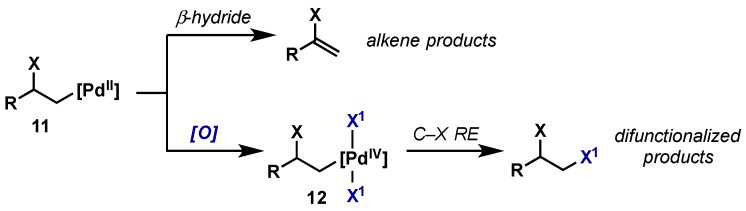
Alkene difunctionalization enabled via Pd(II)/Pd(IV) catalysis.

The difunctionalization of alkenes is also possible employing Pd(II)/Pd(IV) catalysis and hypervalent iodine reagents. Through the traditional Pd(0)/Pd(II) catalysis, the Pd(II) intermediate (**11**) that arises from initial heteropalladation undergoes rapid β-hydride elimination to regenerate an alkene (Scheme 11). By introducing an appropriate oxidant, **11** can instead be oxidized to a Pd(IV) species (**12**), which is set up for subsequent reductive elimination.

**Scheme 12:**
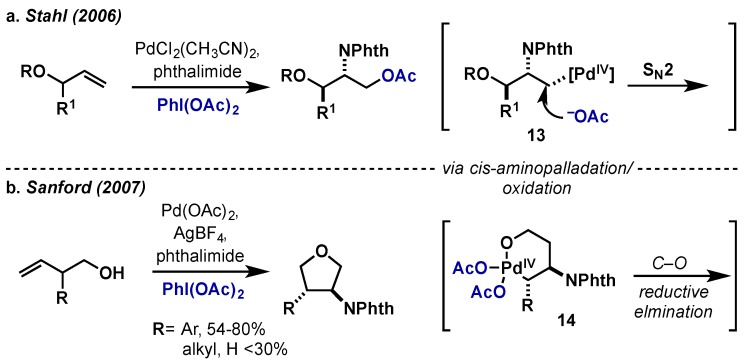
Two approaches to alkene aminooxygenation via Pd(II)/Pd(IV). Changes in substrate lead to a divergence in mechanism for C–O bond formation.

There have been several reports on 1,2-aminooxygenation of alkenes via this approach that utilize phthalimide as the nitrogen source. Stahl reported the use of allylic ethers in the diastereoselective aminoalkoxylation of terminal alkenes (Scheme 12a) [25]. Mechanistic studies revealed that an initial *cis* aminopalladation step and oxidation gave Pd(IV) intermediate **13**, followed by C–O bond formation via an intermolecular S_N_2-displacement by acetate. Using a similar approach, Sanford employed homoallylic alcohols in the diastereoselective formation of substituted tetrahydrofuran rings (Scheme 12b) [26]. Consistent with Stahl’s findings, Sanford reports a *cis* aminopalladation/oxidation sequence however, in this case, the presence of the homoallylic alcohol results in intramolecular coordination to give palladacycle **14**. This leads to preferential direct C–O bond forming reductive elimination rather than intermolecular S_N_2 attack on **14**. Consistent with the necessary formation of the six-membered palladacycle, additional substitution on the alcohol backbone results in significantly higher yields.

**Scheme 13:**
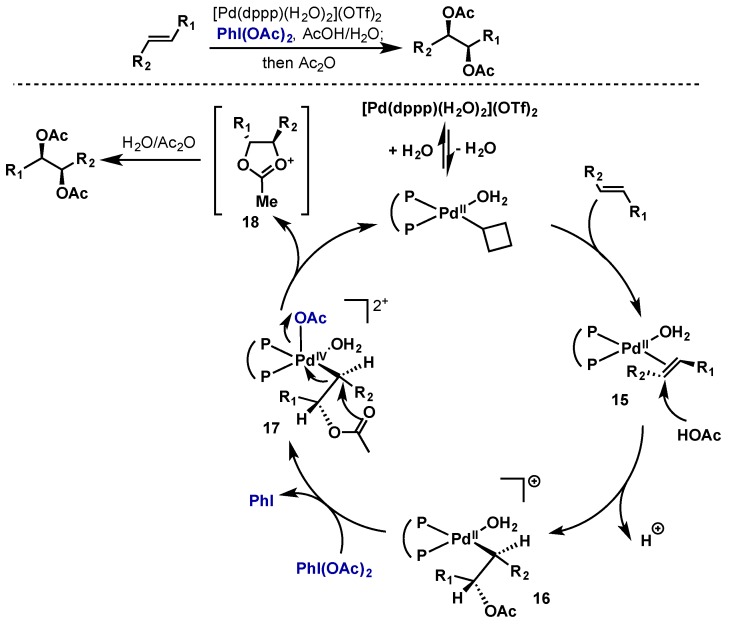
Diastereoselective catalytic alkene dioxygenation using Pd(II)/Pd(IV) catalysis.

Dong reported the dioxygenation of alkenes with [Pd(dppp)(H_2_O)_2_](OTf)_2_ and PhI(OAc)_2_, an attractive alternative to the use of toxic osmium-based reagents (Scheme 13) [27]. Based on labeling studies and the observed *cis* diastereoselectivity, they propose a mechanism involving a Pd(II)/Pd(IV) cycle and an S_N_2-type displacement of a Pd(IV) intermediate. Alkene coordination gives Pd(II) species **15** and promotes intermolecular attack by AcOH to give **16**, which is oxidized by PhI(OAc)_2_ to give Pd(IV) species **17**. **17** then undergoes intramolecular S_N_2-diplacement by acetate giving cyclic oxonium **18** followed by opening with H_2_O and acylation. This method could also be applied in substrates possessing tethered alcohol and carboxylic acid nucleophiles to give tetrahydrofuran and lactone products.

**Scheme 14:**
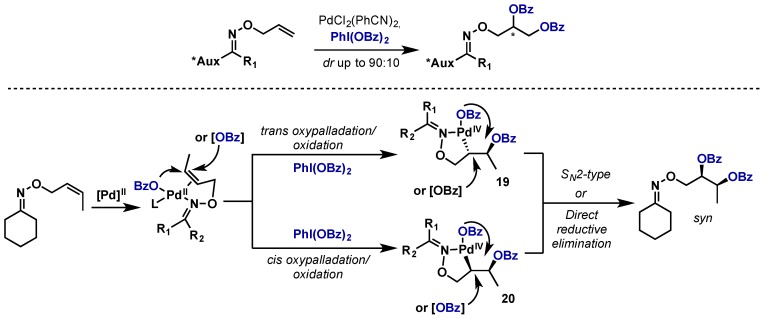
Asymmetric alkene dioxygenation using a chiral oxime directing group.

Using PhI(OBz)_2_ and PdCl_2_(PhCN)_2_, Sanford’s group was also able to perform asymmetric alkene dioxygenation by tethering of a chiral oxime directing group (Scheme 14) [28]. They were able to achieve moderate to high levels of diastereoselectivity on a wide range of oxime substrates possessing different chiral elements. A control experiment using both a *cis* and *trans* alkene showed that both gave rise to their respective *syn* dioxygenated products, shedding some light on the potential mechanism. While the exact pathway was not elucidated, they propose that an initial oxypalladation could occur in either a *trans* or *cis* fashion to give **19** or **20**, each of which can converge to the *syn* product by either an S_N_2-type displacement or direct reductive elimination respectively.

**Scheme 15:**
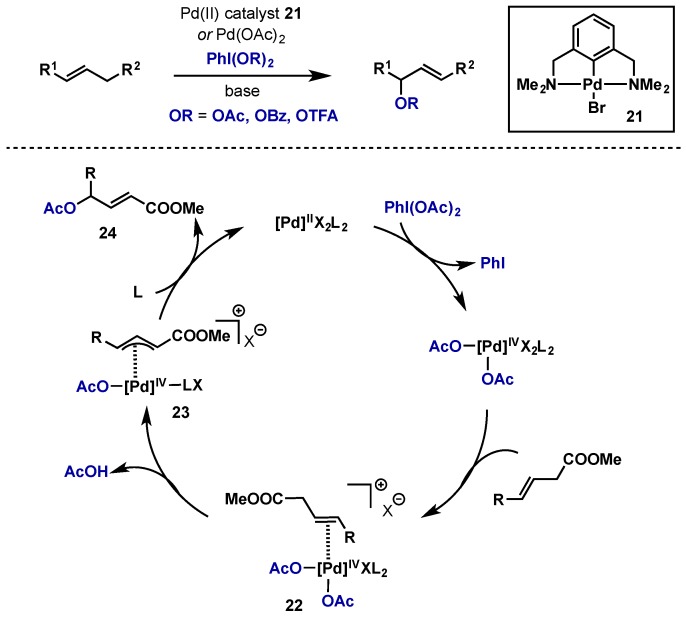
Allylic acetoxylation, benzoxylation, or trifluoroacetoxylation via Pd(II)/Pd(IV) catalysis.

Using a palladium NCN-pincer complex (**21**) or Pd(OAc)_2_, Szabo’s group was able to perform allylic acetoxylation, benzoxylation, or trifluoroacetoxylation using PhI(OAc)_2_, PhI(OBz)_2_, or PhI(OTFA)_2_ (Scheme 15) [29,30]. This offers a complimentary approach to traditional methods of allylic functionalization proceeding via Pd(0)/Pd(II) catalysis. Pd(0)/Pd(II) methods require stoichiometric benzoquinone as an essential additive to activate the allyl Pd(II) species for nucleophilic attack and the use of a more electrophilic Pd(IV) intermediate obviates the need for this activation. The proposed catalytic cycle is shown in the context of acetoxylation, beginning with oxidation of Pd(II) to Pd(IV) and subsequent alkene coordination to give complex **22**. Pi-allyl formation gives **23**, which can then undergo reductive elimination to give desired product **24**. It should be noted that White has reported a Wacker oxidation employing a combination of Pd(OAc)_2_ and catalytic PhI(OAc)_2_, however mechanistic studies indicate that this does not involve direct oxidation to a Pd(IV) intermediate as no ArI byproducts were observed [31].

#### 2.2.3. Carbon-Halogen, Carbon-Boron Bond Formation

One of the most interesting transformations enabled by high valent palladium catalysis is in carbon-halogen and carbon–boron bond formation. In Pd(0)/Pd(II) catalysis, these groups represent functional handles that undergo facile oxidative addition and the reverse process is highly disfavored. Pd(II)/Pd(IV) manifolds offer the perfect compliment, allowing the installation of these valuable atoms into carbon scaffolds via C–H functionalization. The application of hypervalent iodine reagents in this area is limited by the relatively low stability and high reactivity of these reagents that possess halogen ligands. PhICl_2_ is the most common reagent of this type and has seen the most use, more often in high valent complex isolation, whereas *N*-halosuccinimides have dominated synthetic transformations [2].

##### 2.2.3.1. Carbon-Halogen Bond Formation

**Scheme 16:**
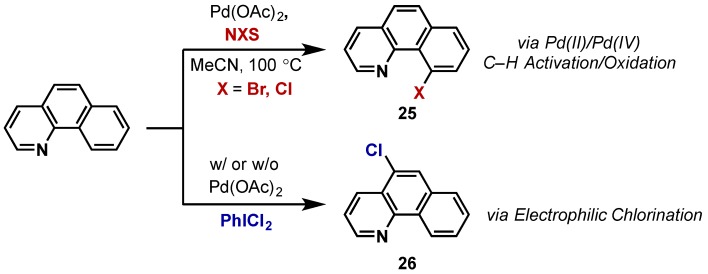
*N*-halosuccinimide oxidants give rise to directed C–H halogenation whereas PhICl_2_ gives product of direct electrophilic chlorination.

In her 2004 report, Sanford shows that the use of *N*-chloro or *N*-bromosuccinimide (NCS, NBS) for the chlorination or bromination of benzoquinoline give rise to the directed halogenation products in good yield (**25**, **26**, Scheme 16) [12]. In contrast, the use of PhICl_2_ gave C5-chlorinated products both in the presence and absence of palladium, indicating a direct electrophilic chlorination mechanism as opposed to a C–H activation pathway. This highlights the disadvantages of using such a highly reactive hypervalent iodine reagent for arene halogenation.

**Scheme 17:**
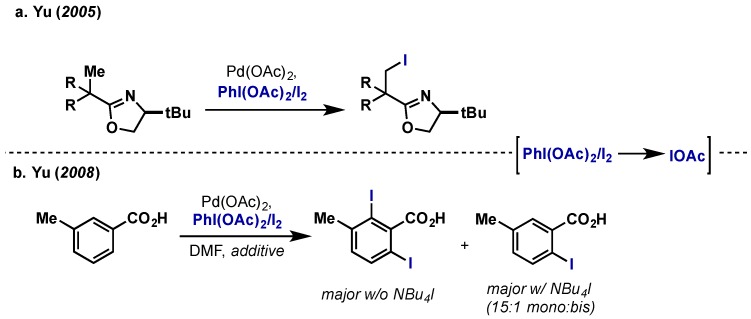
Directed iodination of (**a**) C(sp^3^)–H and (**b**) C(sp^2^)–H bonds using in situ generated IOAc.

There have been several reports of C–I bond formation using the Suarez-type iodine reagent IOAc, often generated via reaction of PhI(OAc)_2_ and I_2_ in situ. Yu initially demonstrated this approach in the directed iodination of unactivated primary C(sp^3^)–H bonds using an oxazoline directing group (Scheme 17a) [32,33]. The use of a chiral oxazoline gave good to excellent levels of diastereoselectivity (91:9 to 99:1) in prochiral substrates. A subsequent report from Yu showed the directed α-iodination of benzoic acids under similar conditions giving rise to predominantly diiodinated products (Scheme 17b) [34]. It was found that DMF was essential for high conversion and the use of tetrabutylammonium iodide as an additive could help control mono- vs. bisiodination products. While the mechanism of Pd(II) oxidation with IOAc has not been fully elucidated, KIE indicate that C–H bond cleavage is proceeding via an electrophilic mechanism in these cases and thus a Pd(II)/Pd(IV) catalytic cycle is proposed.

**Scheme 18:**
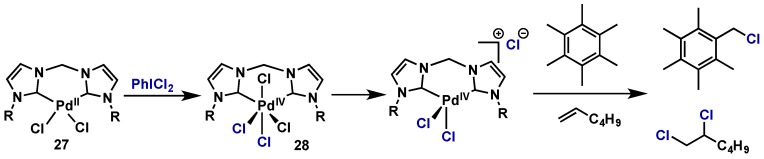
Stoichiometric chlorination of linear alkenes or benzylic positions with a cationic Pd(IV) generated upon oxidation with PhICl_2_.

Kraft demonstrated the use of a Pd(II)-NHC complex for stoichiometric dichlorination of linear alkenes and monochlorination of benzylic C–H bonds (Scheme 18) [35]. PhICl_2_ oxidizes Pd(II) species **27** to Pd(IV) (**28**), and subsequent loss of chloride generates a cationic, pentacoordinated Pd(IV) species that is active for C–H chlorination.

**Scheme 19:**
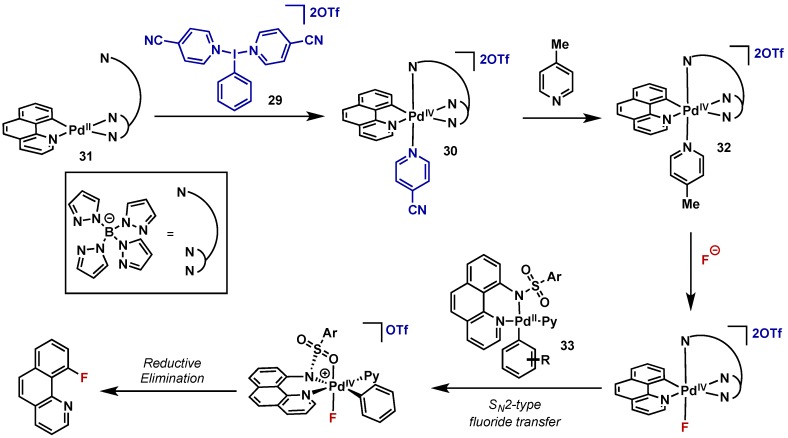
Ritter’s approach to radiofluorination using nucleophilic ^18^F and a (poly)cationic λ^3^-iodane.

Direct C–H fluorination is challenging for transition metal catalysis due to the low reactivity of metal-fluorine bonds towards reductive elimination. Several attempts have been made using Pd(II)/Pd(IV) catalysis, however the use of hypervalent iodine oxidants has been met with challenges. This is due to the high reactivity of the most common λ^3^-iodane X-ligands, namely acetates or halogens, to undergo competitive reductive elimination. In this area, Ritter has been successful in the radiofluorination of C(sp^2^)–H bonds using stoichiometric Pd(II) complexes as fluorinating agents and a (poly)cationic λ^3^-iodane (**29**) as the oxidant (Scheme 19) [36]. These (poly)cationic λ^3^-iodane reagents are relatively underutilized in the synthetic literature, however Dutton has also used these complexes to study high valent palladium and platinum complexes and this work is discussed in Section 2.1. Ritter’s report is an extension of his prior work which utilized the “F^+^” source Selectfluor in the two step fluorination of arylboronic acids with a similar complex, however the use of electrophilic fluorinating agents are not readily translatable to radiolabeling applications [37]. They therefore designed a system employing a highly electrophilic Pd(IV) complex (**30**), could then undergo ligand exchange with a source of nucleophilic ^18^F^−^. Pd(IV) complex **30** was accessed via oxidation of **31** with (poly)cationic λ^3^-iodane **29**; the key feature of this oxidant is the donation of a labile heterocyclic ligand which can undergo facile ligand exchange, first with 4-picoline to give **32**, and subsequently with fluoride upon exposure to a nucleophilic ^18^F-source in the reaction conditions. This complex then serves as a highly electrophilic source of ^18^F for a second Pd(II) species (**33**) which then undergoes C–F bond reductive elimination to give desired product. In a later report they also report the use of an analogous nickel-mediated process under similar conditions (see Section 5.2).

**Scheme 20:**
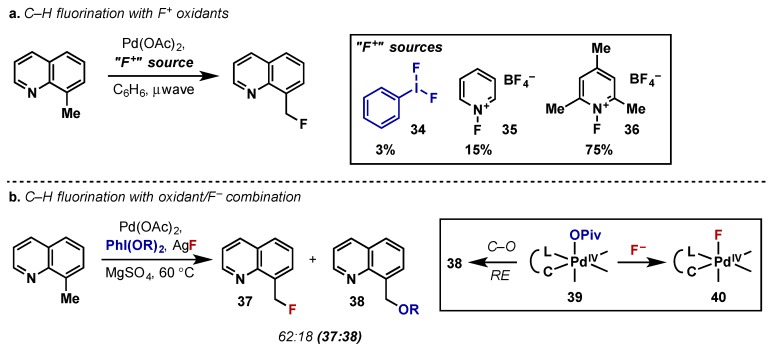
Complimentary approaches to C–H fluorination with Pd(II)/Pd(IV) catalysis via (**a**) electrophilic fluorine sources and (**b**) nucleophilic fluorine sources.

In catalytic Pd(II)/Pd(IV) approaches to C–H fluorination, electrophilic *N*-fluoropyridinium reagents (**35**, **36**) have been much more successful than the analogous hypervalent iodine reagent PhIF_2_ (**34**), as both the oxidant and fluoride source (Scheme 20a) [38,39]. Sanford has attempted to utilize a nucleophilic fluoride source in combination with a hypervalent iodine oxidant achieve catalytic benzylic fluorination (Scheme 20b) [40]. This is a much more economical approach as nucleophilic fluoride sources are considerably less expensive than electrophilic reagents (e.g., **36**—$88,295/mol vs. KF $3.95/mol). A critical challenge to this approach is the relative rates of competitive C–O bond forming reductive elimination at Pd(IV) (**39**) relative to displacement of the carboxylate ligands by F^−^ to give **40**. The use of AgF as the nucleophilic fluoride source proved critical as well as the use of a sterically hindered oxidant PhI(OPiv)_2_ to suppress C–O bond formation, however this pathway could never be completely eliminated. Therefore, while the combination of oxidant and nucleophilic fluoride source is certainly attractive, the choice of oxidant remains challenging and continued research in this area is required.

##### 2.2.3.2. Carbon-Boron Bond Formation

**Scheme 21:**
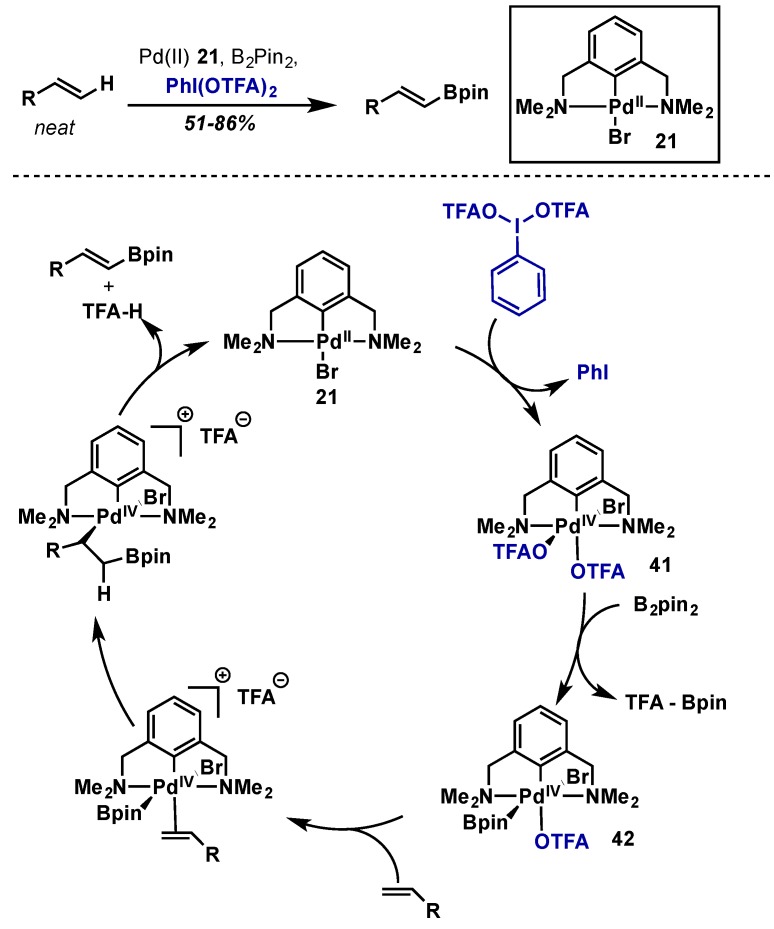
C–H borylation of olefins under oxidative conditions with PhI(OTFA)_2_.

The selective C–H borylation of alkenes under oxidative conditions was reported by Szabo in 2010, giving rise to valuable alkenyl boronates (Scheme 21) [41]. Using an NCN-pincer Pd(II) catalyst (**21**), PhI(OTFA)_2_ as an oxidant, and B_2_pin_2_, the borylation of simple alkenes could be accomplished in good yield. It is noted that a particular advantage of this method is that the oxidizing conditions produce TFAO-BPin upon transmetallation rather than borohydrides, which avoids competitive hydroboration of the resultant alkene products. While the authors could not fully elucidate the mechanism of this process, they propose that initial oxidation of catalyst **21** generates a highly electrophilic Pd(IV) species **41** which then undergoes facile transmetallation with B_2_pin_2_ to give **42**. Alkene coordination, insertion, and finally elimination and decomplexation give the desired products and regenerate **21**. The method is limited by the need to use solvent quantities of the alkene, and internal alkenes react very poorly.

#### 2.2.4. Carbon-Nitrogen Bond Formation

Carbon–nitrogen (C–N) bond formation is a valuable synthetic transformation as nitrogen atoms are ubiquitous is bioactive molecules. Pd(0)/Pd(II) catalysis has served as a valuable approach for the formation of C(sp^2^)–N bonds via the venerable Buchwald-Hartwig amination of aryl halides. Unfortunately wide spread approaches to C–N bond formation, via either C–H activation, alkene functionalization, or others, remains a challenge in palladium catalysis. This is due both to the high activation barrier for C–N bond reductive elimination from Pd(II) species and the fact that many amine substrates will coordinate to palladium, leading to rapid catalyst deactivation. Innovative approaches relying on Pd(II)/Pd(IV) catalysis have emerged to address these challenges and this area has been recently reviewed by Muniz [42]. Hypervalent iodine reagents have played a key role in this area, with careful tuning of oxidant sterics and electronics playing a role in the success a given method.

##### 2.2.4.1. Alkene Diamination

**Scheme 22:**
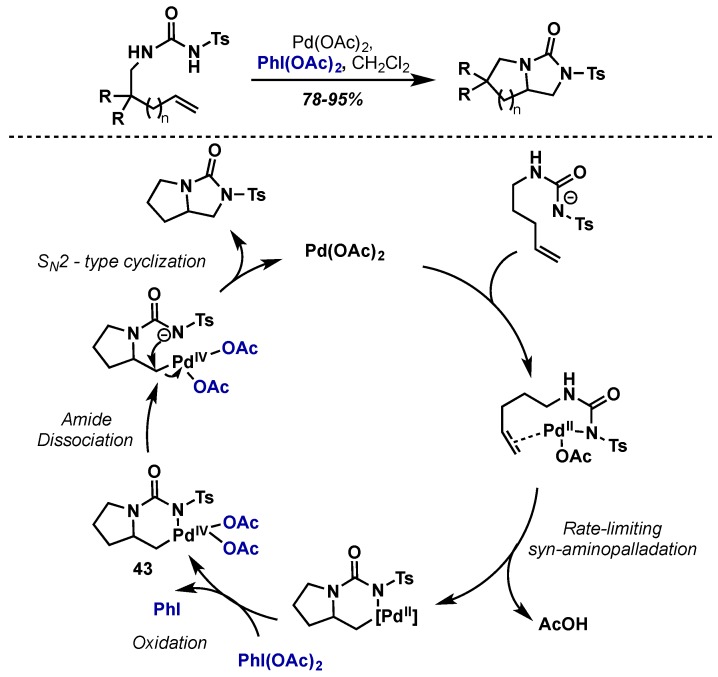
Intramolecular diamination of alkenes via a S_N_2-type displacement of a Pd(IV) intermediate.

The Muniz group has been a leader in the development of both intra- and intermolecular approaches to alkene diamination via Pd(II)/Pd(IV) catalysis [42]. Their first report in this area involved the intramolecular diamination of terminal alkenes with urea derivatives, a particularly challenging transformation due to the high coordinating ability of these substrates to palladium (Scheme 22) [43]. Their method had broad scope and gave the bicyclic 1,2-diamine products in excellent yields. They note that the choice of oxidant was critical and only PhI(OAc)_2_ was able to promote the reaction with high efficiency. A subsequent mechanistic investigation showed the reaction proceeds via rate limiting aminopalladation, followed by oxidation to give Pd(IV) intermediate **43**, which then undergoes S_N_2-type displacement by the second amine [44]. This catalytic cycle is supported by stoichiometric studies conducted in their group (employing PhI(OAc)_2_ as the oxidant), which showed that C–N bond formation proceeded via ligand ionization and subsequent S_N_2-displacement rather than a concerted reductive elimination from Pd(IV) [45]. This general catalytic cycle is invoked for their subsequent applications in both intra- and intermolecular amination reactions.

**Scheme 23:**
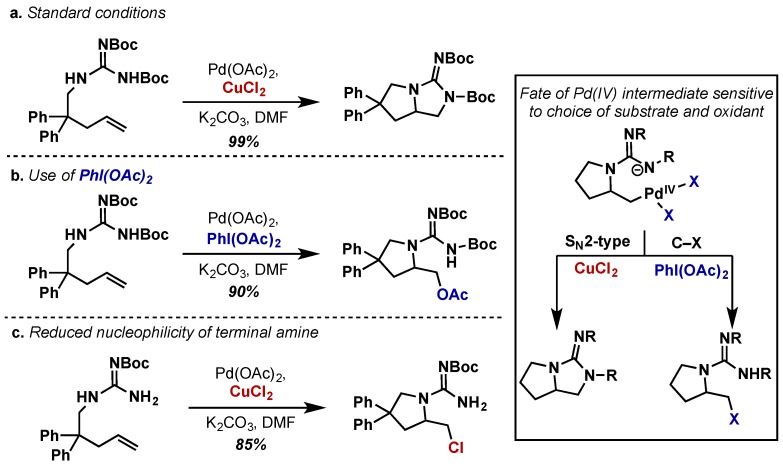
Intramolecular alkene diamination with guanidines. Reactivity of Pd(IV) intermediate sensitive to both substrate and oxidant selection. (**a**) Standard conditions with CuCl_2_ as oxidant (**b**) Use of PhI(OAc)_2_ as oxidant (**c**) Terminal amine with CuCl_2_.

Muniz extended this approach to Boc-protected guanidine substrates, which required a change in oxidant from PhI(OAc)_2_ to stoichiometric CuCl_2_ (Scheme 23a) [46]. In this case, the use of PhI(OAc)_2_, a more powerful oxidant than CuCl_2_, gives rise to exclusively the aminoacetoxylated product as a result of competitive C–O reductive elimination (Scheme 23b). Furthermore, reducing the nucleophilicity of the terminal amine in the presence of CuCl_2_ led to aminochlorinated products (Scheme 23c). This S_N_2 cyclization mechanism is also analogous to Sanford’s Pd(IV) mediated cyclopropanation and these results clearly indicate that the further development of S_N_2 based methods with Pd(IV) will depend on careful tuning of both oxidant and nucleophile [47]. A final report of intramolecular diamination from Muniz involved the intramolecular diamination of stilbene derivatives to yield bisindoline substrates employing Pd(OAc)_2_ and PhI(OAc)_2_ (not shown) [48].

**Scheme 24:**
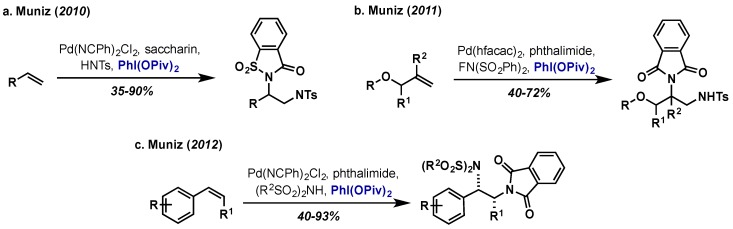
Reports from the Muniz group on intermolecular alkene diamination with PhI(OPiv)_2_ as the oxidant. (**a**) Diamination of terminal alkenes (**b**) Diamination of allyl ethers (**c**) Diamination of styrene derivatives.

Extending this approach to intermolecular diamination was challenging as C–O bond forming reductive elimination formation would be more competitive relative to a slower intermolecular S_N_2 amine displacement. In three reports, the Muniz group succeeded in addressing this challenge in the intermolecular diamination of terminal alkenes, allyl ethers, and finally internal styrene derivatives with phthalimide, saccharide or *N*-fluoro-bis(phenylsulfonyl)imide (NFSI) as the amine sources (Scheme 24) [49,50,51]. In all cases, PhI(OPiv)_2_ was employed as the hypervalent iodine oxidant as PhI(OAc)_2_ gave very low yields with a range of palladium catalysts. The use of the more sterically encumbered PhI(OPiv)_2_ is presumably critical to address the issue of competitive C–O reductive elimination as the –OPiv group will undergo this process much slower than the corresponding –OAc, allowing intermolecular S_N_2 displacement to occur. It is noteworthy than a similar approach from Michael utilized NFSI as both the amine source and external oxidant, circumventing competitive C–X reductive elimination [52,53].

##### 2.2.4.2. C–H Amination

**Scheme 25:**
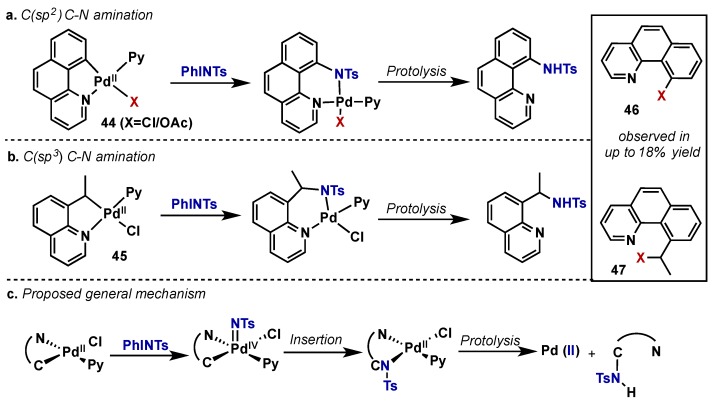
Stoichiometric study of directed C–H bond amination with Pd(II) and PhI=NTs as oxidant and N-source. **Insert**: Byproducts observed as a result of competitive C–X bond formation.

Sanford reported a study of C(sp^2^) and C(sp^3^)–H bond amination using Pd(II) catalysts and PhI=NTs as the oxidant in a stoichiometric context (Scheme 25) [54]. Using various palladacyclic species **44**, **45**, generated via directed C–H activation, they found that C–H insertion happens readily upon oxidation with PhI=NTs. In both cases, byproducts arising from competitive C–X bond forming reductive elimination were observed in up to 18% yield (compounds **46**, **47**), and this was highly dependent on reaction conditions. Oxidant electronics were found to play a role in the rate of C–H insertion; altering the substituents on the benzylsulfonamide PhINSO_2_C_5_H_4_X (X = OMe/NO_2_) led to prolonged reaction times. The authors refrain from drawing mechanistic conclusions from this data due to the highly varied solubility and hydrolytic instability of the hypervalent iodine reagents under the reaction conditions. A general mechanism is shown in Scheme 25c, and the intermediacy of Pd(IV) is supported by the observation of C–X reductive elimination products. Direct C–H activation/amination could also be achieved at sp^3^ C–H bonds, however isolated yields upon protolysis were lower relative to sp^2^ analogues. It should be noted that a catalytic approach to C–H bond amination has been reported using K_2_S_2_O_8_ as a stoichiometric oxidant [55].

**Scheme 26:**
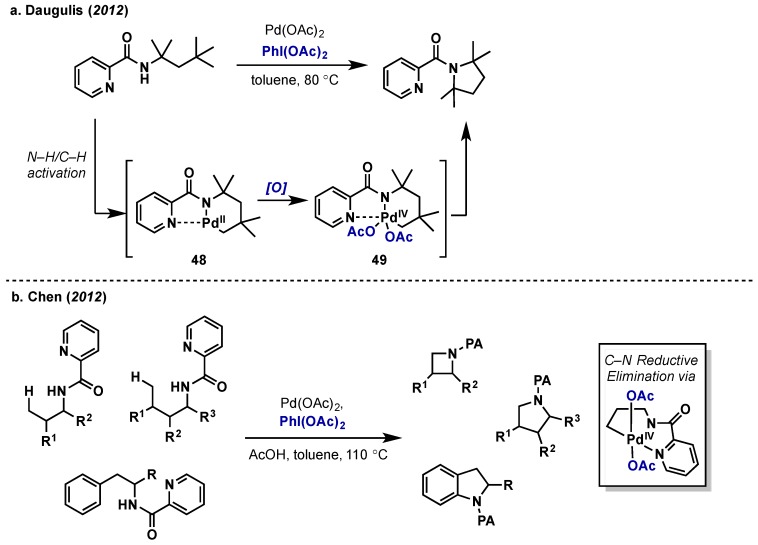
Intramolecular C–H amination via Pd(II)/Pd(IV) catalysis with PhI(OAc)_2_. (**a**) Daugulis report on direct C(sp^3^)-amination (**b**) Chen’s C(sp^3^) and C(sp^2^)–H amination.

Daugulis provided the first report of catalytic alkyl-nitrogen coupling via C–H activation with Pd(II)/Pd(IV), which relied on oxidation with PhI(OAc)_2_ (Scheme 26a) [56]. Other oxidants screened included more electron-deficient λ^3^-iodanes PhI(OTFA)_2_, PhI(*p*-NO_2_C_6_H_4_O_2_C)_2_, as well as AgOAc, all of which gave very low or no conversion. The net intramolecular C–H amination proceeded via consecutive N–H/C–H activation, oxidation of complex **48** to give Pd(IV) intermediate **49**, and final C–N bond forming reductive elimination. Mechanistic insights into the C–N bond forming step were not provided. This work was followed closely by that of Chen who used the picolinamide directing group for the construction of diverse 4- and 5-membered nitrogen heterocycles via C(sp^3^)–H amination (Scheme 26b) [57].

**Scheme 27:**
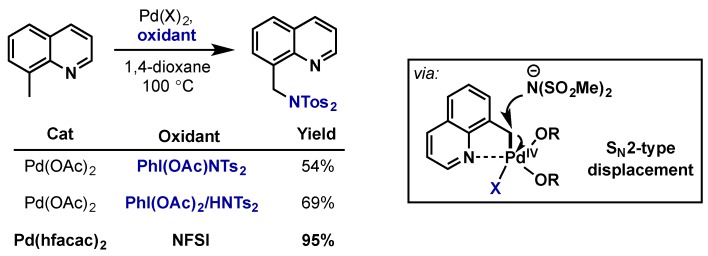
Directed C(sp^3^)-amidation of benzylic C–H bonds via S_N_2-displacement of Pd(IV).

The Muniz group reported a benzylic C–H amidation via a directed C–H bond activation and subsequent S_N_2-type displacement at Pd(IV) (Scheme 27) [58]. In this reaction it was more effective to have the oxidant also serve the source of nitrogen and hypervalent iodine reagents therefore proved to be less efficient than NFSI. A more electron deficient bidentate hexafluoroacetylacetonate (hfacac) ligand on palladium also improved efficiency. This reaction could be extended to anisole and pyridine directing groups, giving a rather versatile method for C–H bond amidation. Despite its utility, the use of NFSI as the oxidant does lead to generation of an equivalent of HF, and thus discovery of more environmentally friendly alternatives would be a significant advancement for large-scale applications.

**Scheme 28:**
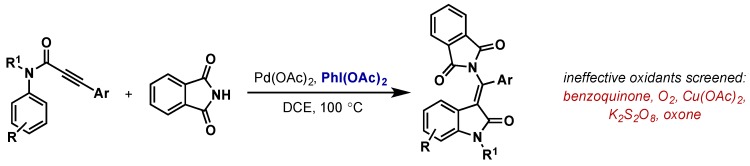
Synthesis of substituted oxindoles via sequential aminopalladation/C–H activation.

Li reported the synthesis of oxindole derivatives via a intermolecular aminopalladation/C–H activation cascade employing Pd(OAc)_2_ and PhI(OAc)_2_ (Scheme 28) [59]. In this case, other oxidants such as oxone, K_2_S_2_O_8_, Cu(OAc)_2_, benzoquinone, and O_2_ were ineffective, giving little or no conversion to products. The authors propose either terminal C–N reductive elimination from Pd(IV) or Pd(IV) C–H activation, however further mechanistic investigation is required.

**Scheme 29:**
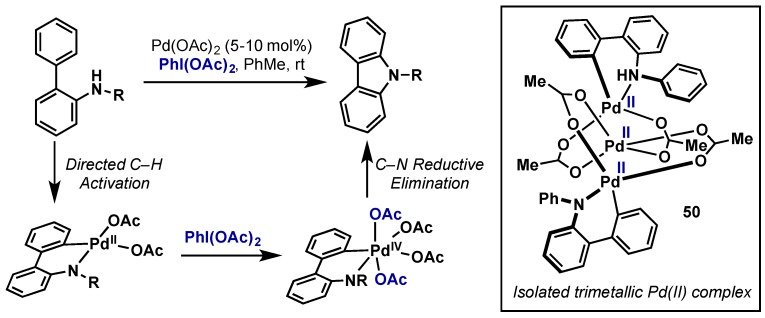
Direct C(sp^2^)-amination at ambient temperature via Pd(II)/Pd(IV).

The synthesis of carbazole derivatives via an oxidative C–H amidation procedure with Pd(OAc)_2_ and PhI(OAc)_2_ at ambient temperature was reported by Gaunt in 2008 (Scheme 29) [60]. This protocol offers an alternative to Ulmann and Buchwald-Hartwig coupling reactions and obviates the need for prefunctionalization. The reaction mechanism should proceed as the other examples demonstrated in this review. The isolation of a trinuclear Pd(II) complex (**50**) having two cyclopalladated aminobiphenyl connected through bridging acetates to a third Pd(II) suggests the oxidation pathways leads to a Pd(IV) that promotes the reductive elimination. However it is also possible that the trimeric complex dissociates into monomeric species prior to the oxidation.

**Scheme 30:**
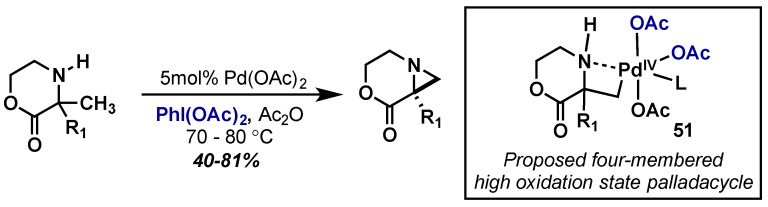
C–H activation approach to aziridines through high oxidation Pd(IV).

Gaunt also reported the synthesis of aziridines through C–H activation with Pd(OAc)_2_ and PhI(OAc)_2_ (Scheme 30). Mechanistic experiments demonstrated that the reaction proceeds through formation of a relatively rare four-membered palladacycle (**51**), which further reductively eliminates to generate the C–N bond. Those experiments also suggest that cyclopalladation is the rate-determining step, followed by fast oxidation by PhI(OAc)_2_. They subsequently translated this to a flow process providing an elegant and efficient approach to the synthesis of challenging strained nitrogen heterocycles [61].

#### 2.2.5. C–C bond Formation

##### 2.2.5.1. Diaryliodonium Salts

For many years, diorganoiodine (III) compounds, represented [Ar–I–R]X^−^, have been utilized for catalytic C–C bond formation via oxidative transfer of “R^+^” to a Pd(0) catalyst and subsequent reductive elimination [62]. In recent years, these reagents have been extended to Pd(II) catalysis, in both stoichiometric and catalytic studies, proceeding via either Pd(III) or Pd(IV) intermediates. From this work, a variety of C(sp^2^–sp^2^) and C(sp^3^–sp^2^) bond formations have been reported.

###### Stoichiometric Studies

**Scheme 31:**
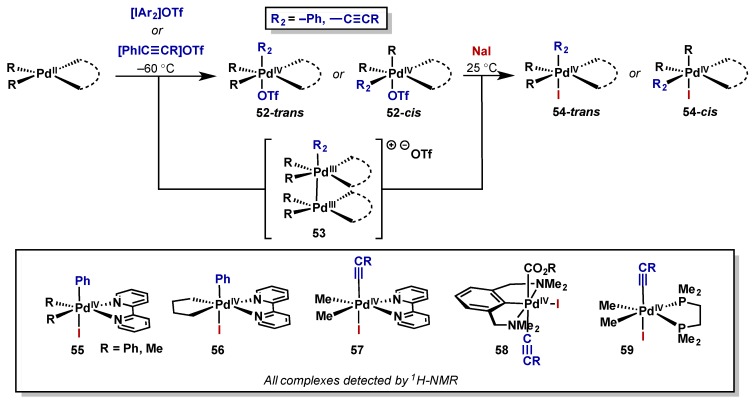
Synthesis of Pd(IV) species upon oxidation with diaryl- and alkynyl iodonium salts.

**Scheme 32:**
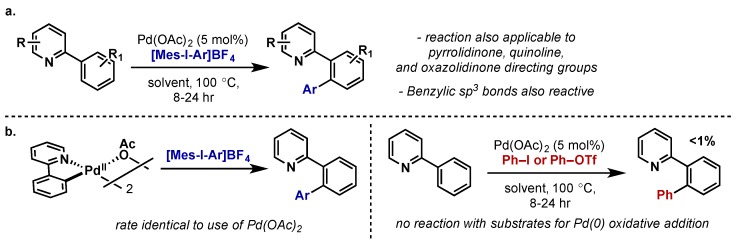
(**a**) Directed C–H arylation with diaryliodonium salts and Pd(OAc)_2_; (**b**) Evidence for Pd(II)/Pd(IV) pathway.

Canty has conducted many of the seminal reports on the isolation of various Pd(IV) and Pt(IV) complexes via oxidation with both aryl and alkynyliodonium salts (Scheme 31) [63,64,65,66]. The iodonium salts were able to cleanly oxidize Pd(II) complexes with a variety of ligand scaffolds, giving rise to varying degrees of *cis/trans* isomers (**52**-***cis*** or **52**-***trans***) and a Pd(III) dimer (**53**), at low temperature. For characterization purposes these complexes were then treated with NaI resulting in triflate displacement or addition into the cationic Pd(III) species (**54**-***cis*** or **54**-***trans***), and a summary of the complexes synthesized via this approach is provided below (**55**–**59**, Scheme 32). Throughout these reports, Canty notes that the palladium complexes are significantly less stable than the corresponding platinum species meaning isolation and characterization were much more challenging. For a further discussion of the analogous platinum components to Canty’s studies, see Section 3.1.

###### Catalytic Applications

In 2005, Sanford reported a directed C–H arylation using diaryliodonium salts and Pd(OAc)_2_, the first report of C–H arylation invoking a Pd(II)/Pd(IV) catalytic manifold (Scheme 32a) [67]. The reaction was applicable to a wide range of heterocyclic directing groups and a range of electron-deficient and electron-rich aryl rings could be transferred in good yields. This method was further extended to benzylic C(sp^3^)–H arylation on C8-methylquinoline as well as the regioselective arylation of 2,5-disubstituted pyrroles (not shown) [68]. This group subsequently reported a variant using polymer-supported iodonium salts that gave equally high yields and the hypervalent iodine reagent could be readily recycled [14]. The reaction was proposed to proceeded via a Pd(IV) intermediate by analogy to their work with C–H acetoxylation (see Section 2.2.2.1), and preliminary mechanistic investigations supported this hypothesis (Scheme 32b). A subsequent study revealed further mechanistic insights, including that oxidation by the diaryliodonium salt was turnover limiting [69]. This is particularly interesting since analogous acetoxylation reaction with PhI(OAc)_2_ are found to be zero-order in PhI(OAc)_2_ and that cyclopalladation is turnover limiting. This implies that oxidation by diaryliodonium salts is much slower than by PhI(OAc)_2_ and this could be significant in the further development of this chemistry as undesirable side reactions could begin to compete with oxidation. A complete, detailed mechanistic study was later conducted between the groups of Sanford, Canty, and Yates, incorporating computational studies, and readers are directed to that report for further discussion [70].

**Scheme 33:**
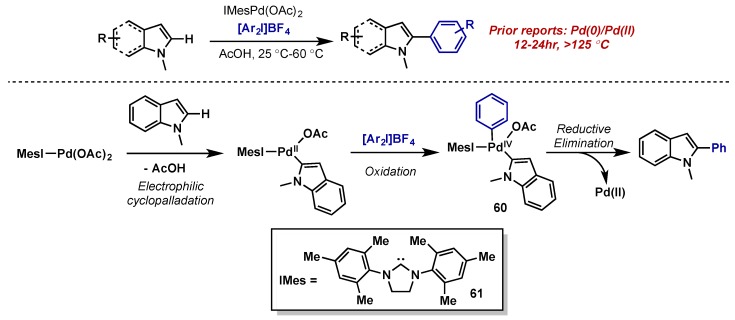
Facile C2-arylation of indoles with diaryliodonium salts through Pd(II)/Pd(IV) catalytic cycle.

In 2006, Sanford reported of the C2-arylation of indoles using diaryliodonium salts (Scheme 33) [71]. The proposed mechanism invoked a Pd(II)/Pd(IV) catalytic cycle beginning with rate-determining cyclopalladation, followed by oxidative addition to give Pd(IV) species **60** and subsequent C–C reductive elimination. This approach improved upon prior reports using Pd(0)/Pd(II), which required long reaction times and high temperatures (12–24 h, >125 °C), by accelerating rate-determining cyclopalladation through use of a Pd(II) catalyst. The use of IMes (**61**) as an ancillary ligand improved conversion by stabilizing the Pd(IV) intermediate, but slowed reaction times, supporting a mechanism wherein electrophilic palladation is rate-limiting.

**Scheme 34:**
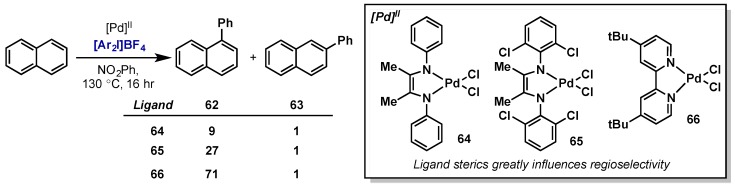
Ligand-controlled regioselectivity in non-directed C–H arylation with diaryliodonium salts.

In a systematic study of ligand effects on regiocontrol in non-directed C–H activation, Sanford examined the C–H arylation of naphthalene with Pd(II) and diaryliodonium salts (Scheme 34) [72]. It was found that subtle changes to ligand sterics had dramatic effects on the regioselectivity of arylation using a range of bidentate diamine ligands. Similar to previous reports, mechanistic investigations revealed oxidation was rate-limiting, however in this case it precedes C–H activation, which then occurs at a Pd(IV) center.

##### 2.2.5.2. Benzoiodoxolones

**Scheme 35:**
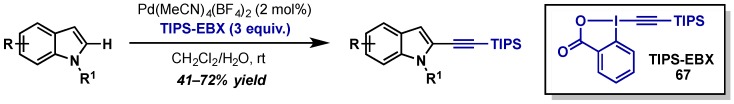
Direct C2-alkynylation of indoles via Pd(II)/Pd(IV) using alkynyl benzoiodoxolone **67**.

In 2013, Waser reported the direct C2-alkynylation of indoles using 1-[(triisopropylsilyl)ethynyl]-1λ^3^,2-benziodoxol-3(1*H*)-one (TIPS-EBX, **67**) as both an alkynyl transfer reagent and oxidant (Scheme 35) [73]. The authors have worked extensively with this reagent with further applications in Au(I)/Au(III) catalysis (see Section 4.3). This method is proposed to proceed through a Pd(II)/Pd(IV) catalytic cycle highly analogous to that proposed for C–H arylation (see previous discussion). This approach addresses issues of C2/C3 selectivity in prior methods and products are obtained in moderate to good yield.

##### 2.2.5.3. Trifluoromethylation

**Scheme 36:**
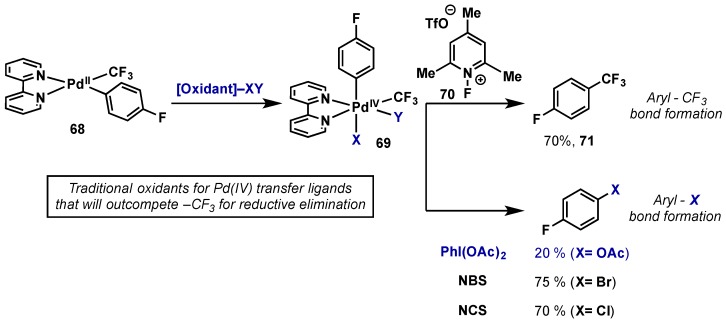
Isolation and study of –CF_3_ reductive elmination from Pd(IV) complexes.

Palladium-catalyzed approaches to arene trifluoromethylation are rare, largely because most palladium (II) species are inert to Ar-CF_3_ bonding forming reductive elimination [74,75,76]. Methods involving a Pd(II)/Pd(IV) cycle are an attractive alternative due to more facile reductive eliminations from high oxidation state Pd(IV). Unfortunately, a limiting factor of most common Pd(IV) oxidants, including hypervalent iodine reagents, is the transfer of X-groups to the metal center that will readily outcompete a –CF_3_ group for reductive elimination. This problem was exemplified in studies by Sanford, wherein the synthesis and stoichiometric reactivity of a Pd(IV)–CF_3_ complex was examined with a range of common oxidants (Scheme 36) [77,78]. Upon oxidation of complex **68**, high oxidation state Pd(IV) intermediate **69** could undergo two possible reductive elimination pathways to form with an Ar–CF_3_ or Ar–X bond. Employing PhI(OAc)_2_ or *N*-halosuccinimides exclusive C–X bond formation was observed, in varying yields. Only upon use of a “F^+^” source as an oxidant, the optimal being NFTPT (**70**), was Ar–CF_3_ bond formation observed in high yield (**71**). This landmark study shows the feasibility of Ar–CF_3_ bond formation via a Pd(IV) intermediate, however it also exposes the limitations of current oxidants. Common “F^+^” sources are expensive relative to hypervalent iodine reagents or *N*-halosuccinimides and their use in catalytic reaction manifolds of this type is not proven. Therefore, the identification of suitable oxidant/ligand combinations that will facilitate selective –CF_3_ reductive elimination from Pd(IV) is of critical importance to the advancement of this area.

**Scheme 37:**
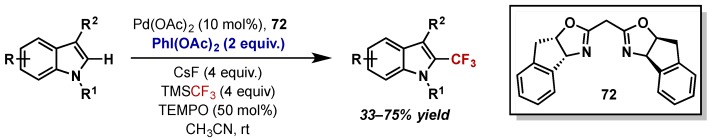
Catalytic trifluoromethylation of indoles with PhI(OAc)_2_ as the oxidant and TMSCF_3_ as the trifluoromethyl source.

In a significant report, the catalytic trifluoromethylation of indoles was reported by Liu, utilizing TMSCF_3_ as the trifluoromethyl source and PhI(OAc)_2_ as a stoichiometric oxidant (Scheme 37) [79]. The authors propose a Pd(II)/Pd(IV) catalytic cycle, involving electrophilic palladation at Pd(II), followed by oxidation by PhI(OAc)_2_/TMSCF_3_ and reductive elimination. However, detailed mechanistic support is not provided and this result is perhaps surprising given Sanford’s previous reports detailing competitive –OAc vs. –CF_3_ reductive elimination at Pd(IV) [77,78]. Given the complex set of reaction conditions employed, a more detailed mechanistic study is required to definitively prove the intermediacy of Pd(IV), and such a study could provide valuable insights for further development of Pd(IV) trifluoromethylation with PhI(OAc)_2_ as an oxidant.

**Scheme 38:**
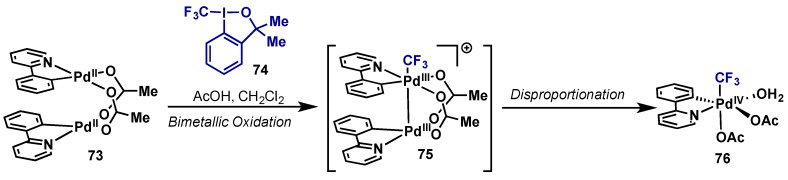
Evidence for intermediacy of bimetallic Pd(III) species en route to monomeric Pd(IV) upon oxidation with Togni’s reagent.

Due to the low reactivity towards reductive elimination, trifluoromethyl palladium complexes have been used as tools to probe mechanistic details and oxidation states of proposed catalytic intermediates. There is an ongoing discussion in the literature whether Pd(IV) monomeric species or Pd(III)–Pd(III) dimers are the intermediates arising from hypervalent iodine oxidation of a Pd(II) species (see Section 2.3.2 for further discussion). Ritter has reported that oxidation of palladium complex **73** with PhI(OAc)_2_ and PhICl_2_ results in the formation of Pd(III) dimers [80]. In contrast, Sanford showed that oxidation of **73** with Togni’s reagent (**74**) gave rise to an isolable Pd(IV) species (**76**) (Scheme 38) [81]. In this study, Togni’s reagent gave the best yield of complex **76**, as compared to other electrophilic “CF_3_^+^” sources. A subsequent study between the groups of Ritter, Yates, Canty, in collaboration with Sanford, showed that monomeric Pd(IV) complex **76** is likely formed through a two-step oxidation/disproportionation sequence, through the intermediacy of bimetallic intermediate **75** [82]. It is suggested that formation of the Pd–Pd bond that occurs during initial oxidation to Pd(III) lowers the activation barrier to oxidation en route to **76**.

#### 2.2.6. Miscellaneous

**Scheme 39:**
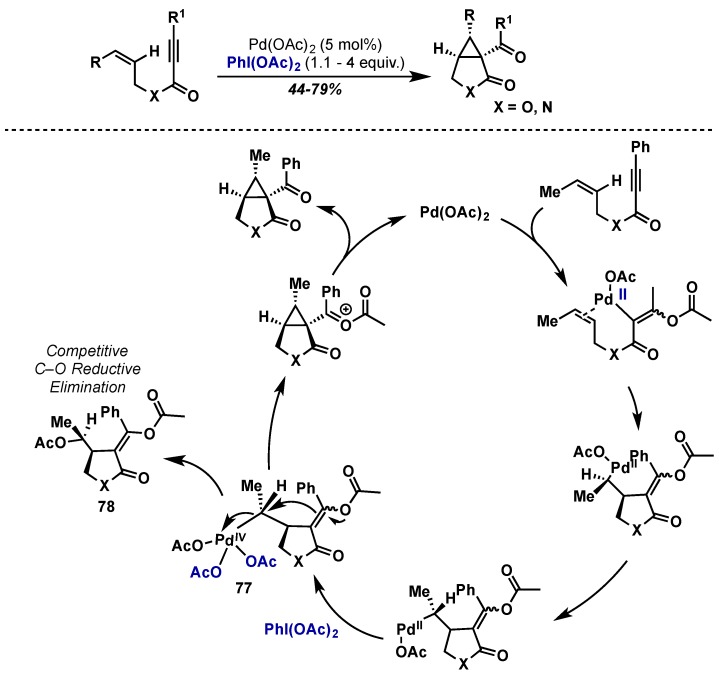
Cyclopropane synthesis via Pd(II)/Pd(IV) catalysis with S_N_2-type displacement of Pd(IV).

Sanford has reported an interesting transformation wherein enynes are converted to highly substituted cyclopropanes via a cyclization cascade employing Pd(OAc)_2_ and PhI(OAc)_2_ (Scheme 39) [47]. In this case, the high-oxidation state of Pd(IV) intermediate (**77**) undergoes nucleophilic displacement by an internal enol ether to generate the cyclopropane moiety via an S_N_2-type pathway, analogous to Muniz’s reports on Pd(IV)-mediated amination (see Section 2.2.4.1). Key to success is that the rate of intramolecular cyclization is faster than that of C–O bond forming reductive elimination from **77** and trace amounts of C–O products (**78**) are observed.

### 2.3. Palladium(III)

#### 2.3.1. Introduction

In contrast to reactions with palladium in its 0, +1, +2, and +4 oxidation states, little is known about the chemistry of Pd(III) dimers or their potential role in catalysis. This has been due to the low stability of these compounds, hindering their isolation and characterization, and a lack of examples employing Pd(III) organometallic species in catalytic manifolds. Over the last 10 years, hypervalent iodine reagents have emerged as excellent oxidants for the isolation of Pd(III) dimers and these studies have provided key insights into the role that Pd(III) intermediates could play in carbo-heteroatom bond forming transformations. In this section we will highlight some of the more significant studies in this area, with an emphasis on those that relate to the previously discussed Pd(II)/Pd(IV) carbon-halogen and carbon-oxygen bond forming reactions.

#### 2.3.2. Complex Isolation

**Scheme 40:**
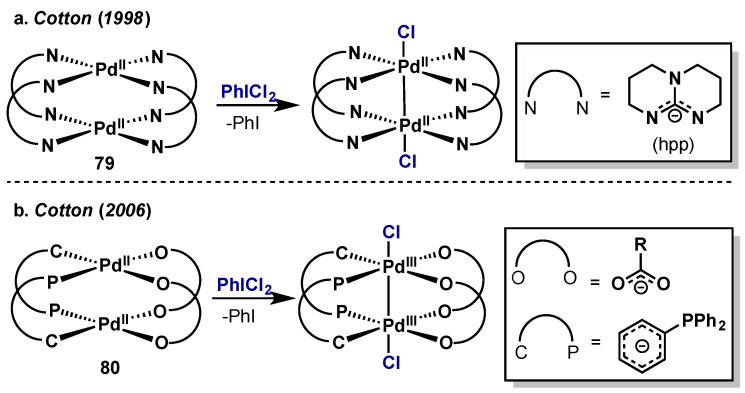
Formation of Pd(III) dimers upon oxidation with PhICl_2_. (**a**) Use of an hpp bridging ligand (**b**) Use of bridging carboxylate and arylphosphine ligands.

Cotton reported the isolation and characterization of several dimeric Pd(III) species possessing a Pd–Pd single bond, all accessed via two, one-electron oxidations of dimeric Pd(II) precursors with PhICl_2_ (Scheme 40) [83,84]. In their initial report, cyclic voltammetry measurements revealed that Pd(II) species **79**, with a bridging hpp (1,3,4,6,7,8-hexahydro-2*H*-pyrimido[1,2–α]pyrimidine) ligand, possessed a quasi-reversible oxidation at –0.12 V and an irreversible oxidation at +0.82 V hinting at the low stability of the products. Indeed, while they were able to achieve oxidation with PhICl_2_, products were isolated in low yield via manual selection of crystals amongst numerous decomposition products. A subsequent study improved on the oxidation by exploring other bridging ligands and found that while it was possible to oxidize Pd(II) dimers with a variety of electron-rich and electron-deficient carboxylate ligands (**80**); oxidation became inaccessible upon introduction of an electron-deficient perfluorinated aryl backbone on the phosphine ligand.

**Scheme 41:**
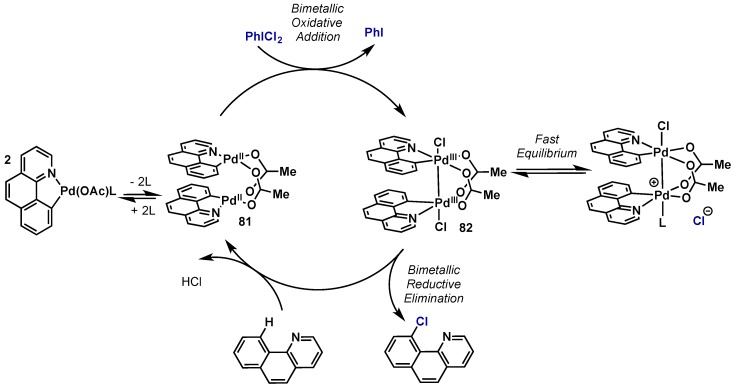
First evidence for the role of bimetallic Pd(III) dimers in catalysis.

Ritter has conducted extensive studies on the potential role that bimetallic Pd(III) dimers could play in what had been widely proposed to be Pd(II)/Pd(IV) catalytic cycles (Scheme 41) [80,82,85]. The first definitive report for the presence of Pd(III) intermediates was in 2009. The oxidation of a dimeric Pd(II) complex (**81**) was examined employing oxidants common to Pd(II)/Pd(IV) C–X bond forming reactions, including PhI(OAc)_2_, PhICl_2_, or *N*-halosuccinimides. Oxidation of **81** with PhICl_2_ gave rise to Pd(III) intermediate **82**, which was characterized by X-ray crystallography. The Pd–Pd bond length in **82** is 2.84 Å, which strongly suggests a metal-metal single bond and bond order of zero. Therefore, in a net two-electron oxidation, PhICl_2_ oxidizes each palladium center by one electron (d^8^ -> d^7^), which results in formation of a metal-metal single bond. Detailed mechanistic and computational studies support that **82** is then able to undergo a concerted reductive elimination event wherein both components of the new C–X bond arise from a single palladium center. A subsequent report from Ritter provides additional evidence that a Pd (III) dimer is the kinetically competent species in the acetoxylation of 2-phenylpyridine with Pd(OAc)_2_/PhI(OAc)_2_ [85].

Together, these reports are the first to show the catalytic competence of Pd(III) dimers in C–X bond forming reactions and could fuel further investigations in the area of bimetallic Pd(III) catalysis. Furthermore it supports the consideration of Pd(III) dimers along Pd(II)/Pd(IV) catalytic cycles employing hypervalent iodine oxidants.

#### 2.3.3. Conclusions

High valent palladium chemistry represents one of the most well developed areas of high oxidation state metal catalysis. Advancements in the formation of a wide range of carbon-heteroatom bonds including C–O, C–X, C–N, as well as C–C bonds via this manifold have been reported. PhI(OAc)_2_ and diaryliodonium salts have been the most widely applied hypervalent iodine reagents in catalytic method development and PhICl_2_ has been successfully used for high valent complex isolation. Detailed mechanistic studies have revealed clear evidence for Pd(II)/Pd(IV) redox couples in these processes, however the role of Pd(III)-dimers along the catalytic cycles is becoming more evident. Current limitations remain in the reductive elimination of challenging groups such as fluoride or trifluoromethyl groups, and development of oxidants that do not possess competitive ligands for reductive elimination would significantly contribute to this area.

## 3. Platinum

**Scheme 42:**
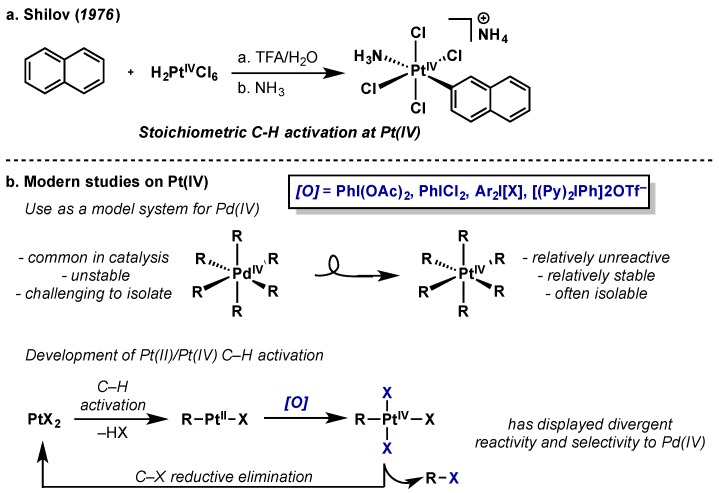
(**a**) Stoichiometric C–H functionalization of naphthalene by Shilov; (**b**) General scheme for modern applications Pt(II)/Pt(IV) redox couples with hypervalent iodine oxidants.

Platinum has played a pivotal role in the evolution of oxidative couplings. Arguably, the “Shilov system” was the first significant example of an intermolecular C–H functionalization, wherein the oxidation of alkanes to a mixture alcohols and alkyl chlorides was mediated by an aqueous solution of [PtCl_4_^2−^] and [PtCl_6_^2−^] (Scheme 42a) [86]. Since this seminal discovery, extensive studies have been conducted on the Pt(II)/Pt(IV) redox couple, as well as the potential formation of Pt(III) dimeric species. Oxidation of square planar Pt(II) to octahedral Pt(IV) is significantly more facile than palladium (standard reduction potentials of [PtCl_6_^2−^] and [PdCl_6_^2−^] are +0.68 V and +1.29 V, respectively) [86], however, Pt(II)/Pt(IV) mediated oxidative couplings are comparatively rare. This is due to the significantly higher barrier to reductive elimination for Pt(IV) relative to Pd(IV) [87]. The enhanced stability of Pt(IV) complexes has been exploited in their use as isolable model systems for the study of more elusive Pd(II)/Pd(IV) redox couples [88]. Mechanistic insights provided by these studies have been recently reviewed [88,89] and this section will cover recent advancements in Pt(IV) chemistry as they relate to hypervalent iodine reagents.

### 3.1. Complex Isolation

**Scheme 43:**
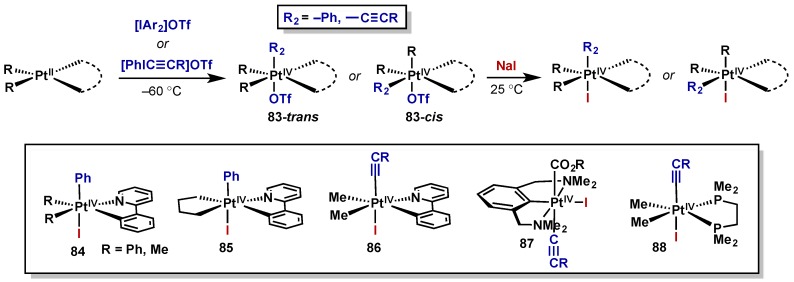
Characterization of Pt(IV) upon oxidation with diaryl and alkynyliodonium salts.

Canty has conducted many of the seminal reports on the isolation of various Pt(IV) and Pd(IV) complexes via oxidation with both aryl and alkynyliodonium salts (Scheme 43) [63,64,65,66]. The iodonium salts were able to cleanly oxidize Pt(II) complexes with a variety of ligand scaffolds, giving rise to varying degrees of *cis/trans* isomers (**83-*cis*** or **83-*trans***), at low temperature. For characterization purposes these complexes were then treated with NaI resulting in triflate displacement, and a summary of the complexes synthesized via this approach is provided below (compounds **84**–**88**). Throughout these reports, Canty notes the higher stability of the resultant platinum complexes relative to palladium, facilitating isolation and structural characterization that was not possible with the corresponding palladium species. For a further discussion of the analogous palladium components to Canty’s studies, see Section 2.2.5.1.

**Scheme 44:**
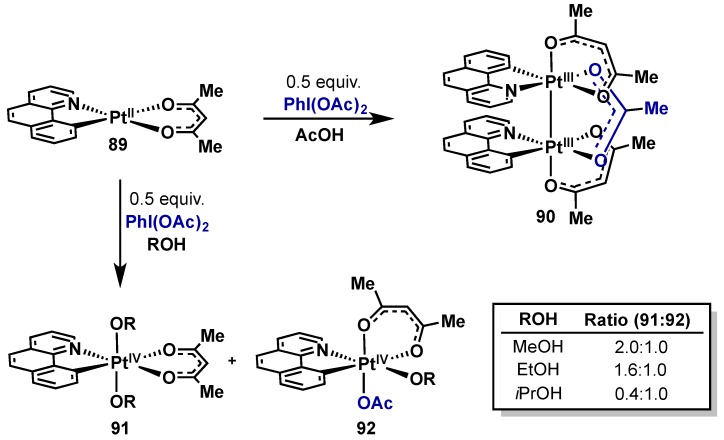
Benzo[*h*]quinoline Pt(II) oxidation studies. Effect of solvent on oxidation product ratios.

In 2005, Sanford reported the oxidation of benzo[*h*]quinoline supported Pt(II)acac complex **89** with PhI(OAc)_2_ in an effort to gain mechanistic insights into the analogous Pd(II)/Pd(IV) system (Scheme 44) [90]. Interestingly, it was found that solvent had a pronounced effect on product distribution. In AcOH, Pt(III)–Pt(III) dimer **90** was obtained, while a mixture of Pt(IV) alkoxides (**91**, **92**) were obtained in the alcoholic solvents, with ratios dependent on the steric bulk of the alcohol. Alcoholic solvents are not able to serve as bridging ligands, thus resulting in preferential formation of the monomeric species (**91**, **92**) and such complexes were hypothesized to be analogous to intermediates in Pd(II)/Pd(IV) C–H oxygenations. In contrast, isolation of Pt(III)–Pt(III) **90** was surprising as these intermediates had not been invoked in Pd(II)/Pd(IV)-catalyzed processes, however subsequent studies from Ritter, Sanford, and others have shown the viability of Pd(III) dimers in these processes (see Section 2.3). Only 0.5 equivalents of PhI(OAc)_2_ were needed for formation of either the monomeric or bimetallic species, providing strong evidence that both pathways proceed via two-electron oxidation.

**Scheme 45:**
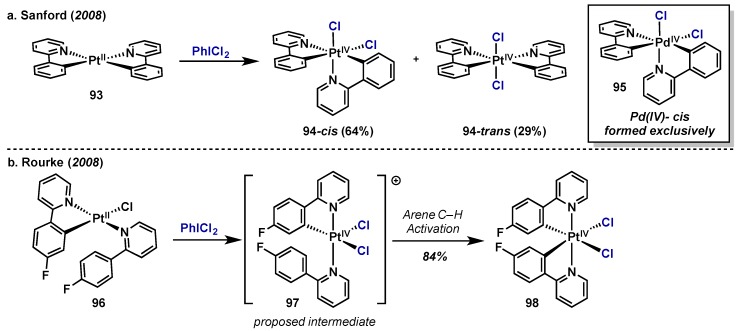
(**a**) Oxidation of 2-phenylpryidine Pt(II) complex with PhICl_2_. Divergent complex geometry from Pd(II); (**b**) Pt(II) oxidation with PhICl_2_ resulting in arene C–H activation.

**Scheme 46:**
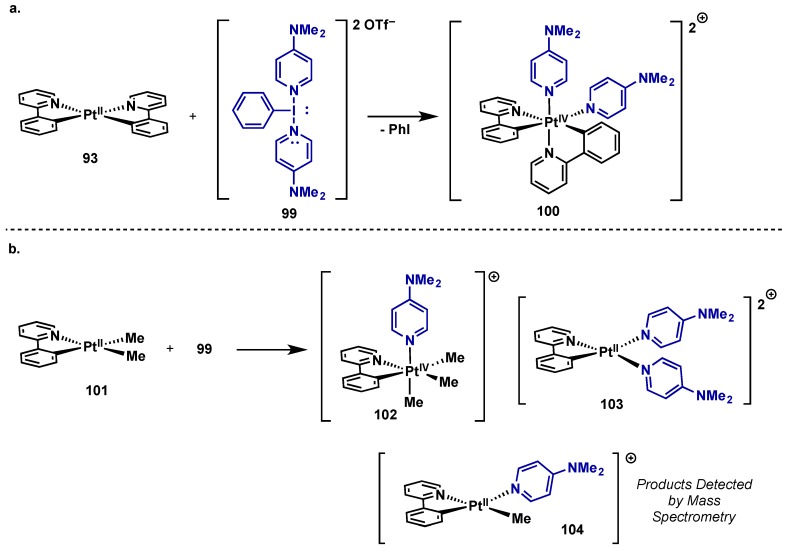
Dicationic [PhI(4-DMAP)_2_][OTf] mediated oxidation of (**a**) 2-Phenylpyridine Pt(II) complex **93**; (**b**) Dimethyl Pt(II) complex **101**.

A subsequent study by Sanford examined the oxidation of 2-phenylpyridine Pt(II) complex **93** with PhICl_2_, which led to a mixture of *cis* and *trans* Pt(IV) complexes (**94-*cis* 94-*trans***, Scheme 45a) [91]. This was particularly interesting as the analogous Pd(II) complex has been found to give exclusive formation of the *cis* isomer (**95**) upon oxidation with PhICl_2_. Pt(II) complex **93** was also subject to a delicate interplay between Pt(IV) and Pt(III)–Pt(III) dimer formation upon oxidation, similar to their previous report [90], however in this case product ratios were contingent on choice of external oxidant. Whereas PhICl_2_ gave exclusively Pd(IV) monomers, altering the oxidant to NCS provided a Pt(III)–Pt(III) dimer as the major product (not shown). Rourke and co-workers showed that treatment of Pt(II) complex **96** with PhICl_2_ resulted in two-electron oxidation with concomitant C–H activation to provide Pt(IV) dichloride **98** even at temperatures as low as −40 °C (Scheme 46b) [92]. This result is notable as previous studies employing other oxidants (peroxides and molecular oxygen) produced complex mixtures. It is proposed that oxidation proceeds via a five-coordinate, cationic Pt(IV) intermediate (**97**) that is highly active towards arene functionalization.

Building on Ritter’s use of poly(cationic) λ^3^-iodanes in high oxidation state nickel and palladium-mediated fluorination (see Section 2.2.3.1 and Section 5.3), Dutton investigated the potential of poly(cationic) λ^3^-iodanes to access a range of dicationic Pd(IV) and Pt(IV) complexes through the delivery of neutral heterocyclic ligands to the metal center (Scheme 46) [93]. They found that 2-phenylpyridine Pt(II) complex **93** could be cleanly oxidized to Pt(IV) complex **100** with a DMAP-derived poly(cationic) λ^3^-iodane **99** (Scheme 46a). However, oxidation of dimethyl Pt(II) **101** led to a less defined product distribution (Scheme 46b), possibly arising from oxidative disproportionation. This reactivity has been documented in similar Pt(II)/Pt(IV) redox couples, however the intermediacy of Pt(III) intermediates cannot be ruled out in this case [94]. These oxidations were also performed on the analogous palladium complexes, which were found to be too unstable for isolation and underwent rapid disproportionation. While the Pt(IV) species **100** and **102** were isolable, it is notable that they are considerably less stable than complexes possessing anionic chloride or acetate ligands, and similar stability trends were observed for the palladium complexes. While this is detrimental in the context of complex isolation, it could be adventitious for enhancing the reactivity of both high-oxidation state palladium and platinum species in catalysis.

### 3.2. Catalytic Applications

While platinum is most commonly employed as a model system, recent advancements have shown its viability in catalytic manifolds. Particularly interesting is the finding that platinum-catalyzed processes often display divergent reactivity and selectivity to those mediated by palladium.

**Scheme 47:**
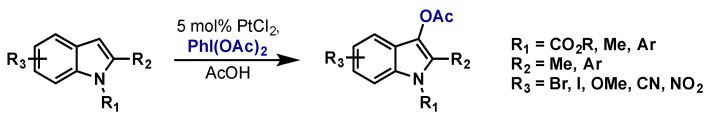
PtCl_2_/PhI(OAc)_2_ mediated C-3 acetoxylation of indoles.

Suna reported that PtCl_2_ with PhI(OAc)_2_ cleanly acetoxylated the C-3 position on various indoles (Scheme 47) [95]. Pd(OAc)_2_ was also a competent catalyst, however PtCl_2_ was found to be more efficient, giving cleaner reactions and higher isolated yields. Other oxidants including K_2_S_2_O_8_, *m*-CPBA, *t*-BuOOH, and Cu(OAc)_2_ were all completely ineffective (0% conversion) and Mg peroxyphthalate gave a complex mixture of products. While not reported, oxidation to the active Pt(IV) species would likely proceed through intermediates similar to those shown in Scheme 43.

**Scheme 48:**
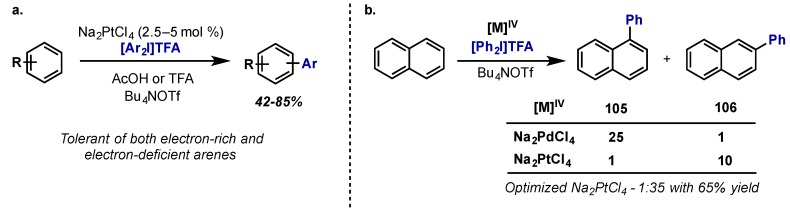
(**a**) Intermolecular C–H arylation with Pt(II) and diaryliodonium salts; (**b**) Reversal of site selectivity for Pt(II) vs. Pd(II) in arene C–H activation.

In 2013, Sanford provided the first example of an intermolecular C(sp^2^)–H arylation enabled by a Pt(II)/Pt(IV) manifold (Scheme 48a) [96]. The reaction was found to have a much broader scope than the analogous palladium-catalyzed transformations, being tolerant of a wide range of both electron rich and electron deficient arenes, and furthermore, a complete reversal in site-selectivity was observed when using Na_2_PtCl_4_ versus Na_2_PdCl_4_ (Scheme 48b). The proposed mechanism proceeds via a Pt(II)/Pt(IV) redox cycle with two-electron C–C bond forming reductive elimination, analogous to previous work on Pd(IV) (see Section 2.2.5) [72].

### 3.3. Conclusions

The true strength of the Pt(II)/Pt(IV) redox couple remains in its use as a model complex for more reactive Pd(II)/Pd(IV) species due to their increased stability. However, the potential synthetic utility of platinum catalyzed reactions is evident, having displayed enhanced reactivity, and divergent selectivity relative to palladium. Dutton’s findings that oxidation employing poly(cationic) λ^3^-iodanes produce less stable Pt(IV) centers relative to traditional oxidants may lead to more reactive Pt(IV) intermediates and expand their applications in oxidative couplings.

## 4. Gold

### 4.1. Introduction

**Scheme 49:**
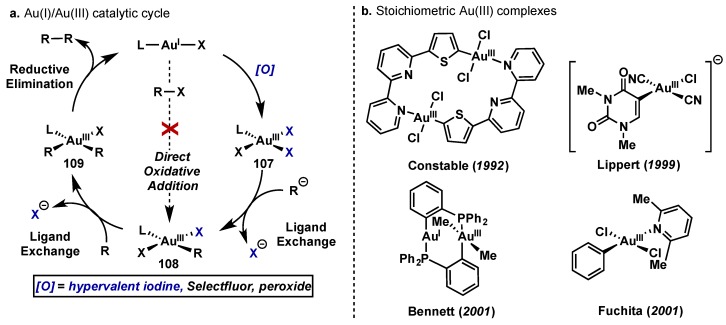
(**a**) Plausible Au(I)/Au(III) catalytic cycle based on use of an external oxidant; (**b**) Pioneering examples of stoichiometric Au(III) complexes.

Gold catalysis has historically proceeded through redox neutral pathways relying on its high efficiency as a carbophilic pi acid. This has seen wide application in the activation of alkynes and alkenes for nucleophilic attack and cycloisomerization cascades, and synthetic applications of these pathways have been recently reviewed [97,98,99,100]. Reactions containing Au(I)/Au(III) redox cycles are rare by comparison, a consequence of the high barrier for oxidation of Au(I) to Au(III) (redox potential +1.41 V). Typical oxidative addition/reductive elimination, which are ubiquitous in Pd(0)/Pd(II) chemistry, are thereby challenging with Au(I)/Au(III) (Scheme 49a, **107** to **109**) [101,102] and examples of such reactivity are scarce [103,104,105]. Instead, Au(III) complexes can be accessed via exposure of an Au(I) species, L_n_–Au–X, to a powerful external oxidant, and in this context hypervalent iodine reagents have found widespread use, along with hydroperoxides, Selectfluor, and others. A catalytic cycle based on this approach is shown in Scheme 49a; oxidation of L_n_–Au–X gives Au(III) species **107**, followed by two subsequent ligand exchanges to access **109**, which would then undergo rapid reductive elimination [102]. This strategy has led to developments in alkynylation, olefin functionalization, cross-couplings and dimerization/homo-coupling reactions using a wide range of oxidants beyond hypervalent iodine species and these reports have been recently reviewed by Gouverneur [102].

Unfortunately, advancements in reaction development centered on Au(I)/Au(III) have not coincided with equivalent mechanistic understanding of the oxidation/reduction pathways or speciation of the organometallic gold complexes involved. Oxidation potentials of active Au(I) species are varied, and can depend on both the counterion and the ligand sphere, making proper oxidant selection delicate and the generation of isolable Au(III) species challenging and unpredictable. However, pioneering studies by Hashmi [106,107], along with the synthesis and characterization of stoichiometric Au(III) complexes by Bennett [108], Lippert [109], Fuchita [110], and Constable [111], have laid the foundations for a more in depth understanding of the chemistry of these highly reactive intermediates (Scheme 49b).

### 4.2. Complex Synthesis and Characterization

Au(I) cationic salts, along with phosphine and N-heterocyclic carbene (NHC) supported Au(I) complexes, are the most utilized in Au(I)/Au(III) catalysis. NHC–Au(I) chemistry in particular has gained immense popularity in the last decade, making them arguably the most well studied of Au(I) complexes. The strong σ–donation of the NHC ligand aides in stabilization of both the Au(I) and Au(III) species, making these complexes ideal candidates for the synthesis of isolable Au(III) complexes [112].

**Scheme 50:**
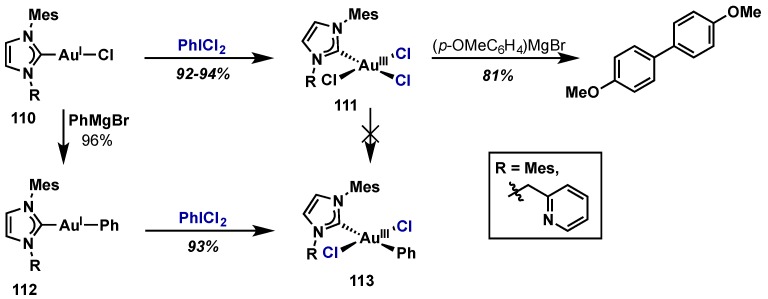
Clean oxidation of NHC Au(I) complexes with PhICl_2_ by Nolan and Limbach.

Limbach and Nolan reported the synthesis of a range of NHC-Au(III) complexes via oxidation of an NHC–Au(I)–Cl with PhICl_2_ (Scheme 50, **110 → 113**) [113,114]. The oxidation could also be carried out with Cl_2_*(g)* at cryogenic temperatures, however the oxidation with PhICl_2_ proceeded more cleanly, in higher yield, and at room temperature, making it far more advantageous. This advantage was attributed to the relatively milder oxidizing conditions when using PhICl_2_ versus Cl_2_*(g)*. Interestingly, attempted oxidations with other λ^3^-iodanes such as Ph_2_IBr, PhI(OAc)_2_, and PhI(OTFA)_2_ either failed to oxidize the Au(I) complexes or gave complex product mixtures. The authors assert that chlorine ligands are crucial to stabilize the Au(III) center and thus PhICl_2_ is optimal as it acts as both an oxidant and chlorinating agent. NHC–Au(I)–Ph complex **112** was also readily oxidized in high yield with PhICl_2_ to give **113**, which the authors state is the first reported Au(III) complex of the type [AuArCl_2_L] [113]. It is worth noting that trichloride Au(III) complex **111** could not be converted to **113** via transmetalation with *p*-methoxyphenylmagnesium bromide, instead giving rise to 4,4′-bismethoxybiphenyl via either a radical coupling or inner-sphere reductive elimination pathway.

**Scheme 51:**
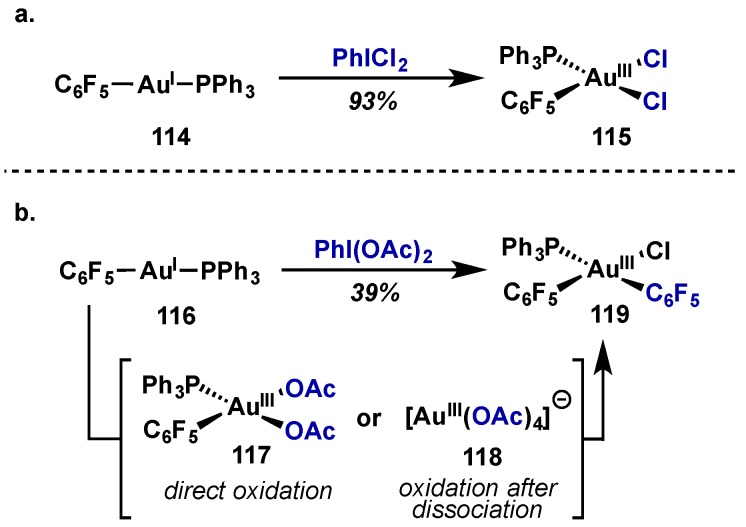
(**a**) Oxidation of [Au(I)(C_6_F_5_)PPh_3_] with PhICl_2_; (**b**) Oxidation of [Au(I)(C_6_F_5_)PPh_3_] with PhI(OAc)_2_.

It has also been shown by Nevado that Au(III) diacetate complexes are less stable than the analogous Au(III) dichloride complexes (Scheme 51). Upon oxidation with PhICl_2_, Au(III) dichloride complex **115** is isolated in high yields (Scheme 51a), however use of PhI(OAc)_2_ leads to isolation of Au(III) bispentafluorophenyl complex **119**, via Au(I) mediated transmetalation (Scheme 51b) [115]. Ligand exchange is proposed to occur through two possible pathways, either via oxidation of Au(I) species **116** to give **117** or of a dissociated [Au(PPh_3_)_2_]^+^ species to give **118**. Both of these complexes would then converge to give **119**, where the chloride atom (observed in X-ray crystallography) is proposed to come from solvent activation.

Based on these findings, PhICl_2_ has become the reagent of choice for the generation of isolable Au(III) complexes, particularly in the context of NHC complexes [112,116,117]. A noteworthy report from Huynh and co-workers utilized PhICl_2_ as the oxidant in a thorough investigation into the structural and electrochemical properties of a range of mono- and bis-NHC Au(I) and Au(III) complexes. This report is an excellent source for data on how oxidation state, NHC, and halide ligands effect the properties of NHC-Au species, and the reader is directed there for a detailed discussion of their findings [112].

**Scheme 52:**
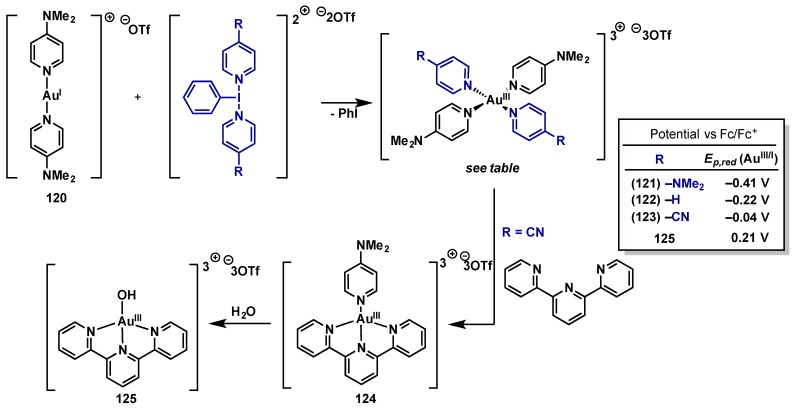
Dutton’s synthesis of tricationic Au(III) complexes and evaluation of their electrochemical properties.

Dutton and co-workers recently utilized (poly)cationic λ^3^-iodanes as neutral ligand-donor oxidants to access tricationic Au(III) complexes [118]. The same group has used these oxidants for the study of Pd(IV) and Pt(IV) complexes (see Section 3.1) and Ritter also employed these reagents as oxidants in a high profile study on Pd(IV)-catalyzed C–H fluorination (see Section 2.2.3.1). Oxidation of Au(I) complex **120** with three different (poly)cationic λ^3^-iodanes possessing varied pyridine ligands resulted in complexes **121**–**123** (Scheme 52). This discovery is significant as prior attempts to access similar complexes via salt metathesis on halogenated Au(III) intermediates led to complex decomposition, emphasizing the power of halide-free external oxidants in Au(I)/Au(III) redox chemistry [117]. Cyclic voltammetry reveals Au(III)/Au(I) reduction potentials ranging from −0.41 to −0.04 V vs. [Fc\Fc^+^] for **121**–**123** (reference 0.069 V vs. [Fc\Fc^+^] for [Au(III)/Au(I)][(dppe)_2_]), showing a trend that mirrors the electron donating ability of the different pyridine ligands ((**121**)NMe_2_ > (**122**)H > (**123**)CN) and highlighting the potential to tune the reactivity of tricationic Au(III) complexes by varying the heterocyclic ligands. Facile ligand exchange from **123** was demonstrated with 2,2,2-tripyridine to give **124**, which could undergo subsequent exchange with H_2_O to give Au(III)–OH complex **125**. The synthesis of terminal Au(III)–OH complexes is rare and previous complexes were accessed via salt metathesis with AgClO_4_ [119]. Intriguingly, homoleptic Au(III) complex (**121**) is stable to aqueous conditions, unlike Au(III) complexes (**122** and **123**). This distinction in reactivity also indicates that chemoselective ligand exchange could be possible in these Au(III) trications.

### 4.3. Synthetic Applications

**Scheme 53:**
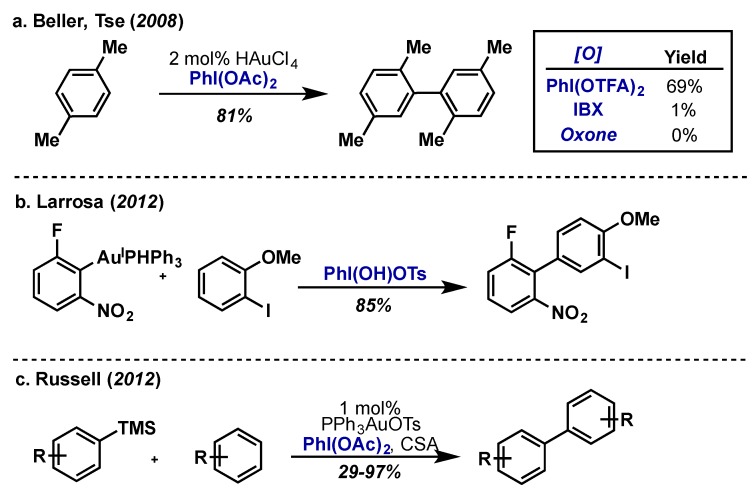
(**a**) First example of a gold catalyzed homo-coupling by Tse and Beller; (**b**) Stoichiometric Au(I)-arene hetero-coupling of unactivated arenes using Koser’s reagent; (**c**) Gold catalyzed arylation of silylated arenes with electron-rich and electron-poor arenes by Lloyd-Jones and Russell.

In 2008, Beller and Tse developed the first gold catalyzed homo-coupling of arenes using HAuCl_4_ with PhI(OAc)_2_ as the external oxidant (Scheme 53a) [120,121]. Mechanistic insights hint that Au(III) is the active C–H functionalization catalyst and that a free radical cation is not likely. PhI(OAc)_2_ showed the highest conversion compared to other oxidants including PhI(OTFA)_2_, IBX and Oxone and subsequent studies found it was essential that the external oxidant be hypervalent iodine derived [121]. More recently, Larrosa and co-workers demonstrated that electron deficient arene-Au(I) species are capable of mediating hetero-coupling reactions with unactivated, electron-rich arenes utilizing Koser’s reagent, PhI(OH)OTs, as the external oxidant (Scheme 53b) [122]. PhI(OPiv)_2_ also gave very high yields in this transformation, however “F^+^” based oxidants Selectfluor and XeF_2_, as well as other acetate-ligated hypervalent iodine reagents (PhI(OAc)_2_, PhI(OTFA)_2_), were ineffective, again emphasizing the delicate nature of oxidant selection in high-valent metal catalysis. It has also demonstrated that silylated arenes are capable of undergoing arylation with a range of electron-deficient and electron-rich arenes under Au(I)/Au(III) redox conditions, using an in situ formed oxidant from PhI(OAc)_2_ and camphor sulphonic acid (CSA) (Scheme 53c) [123]. Other carboxylate ligands of the PhI(O_2_CR)_2_ type were also effective, however, λ^3^-iodane iodosylbenzoic acid and the “F^+^” oxidant Selectfluor were completely ineffective, producing none of the desired product.

**Scheme 54:**
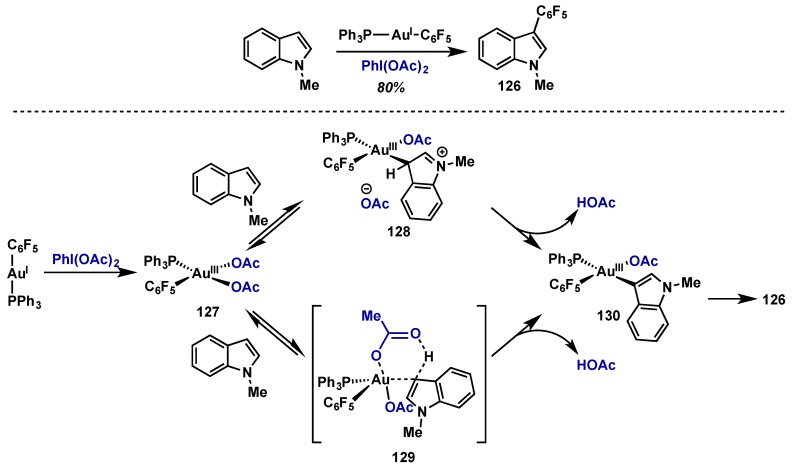
Proposed mechanism of Au(I)/Au(III) catalyzed hetero-coupling of electron rich arenes.

Nevado recently provided mechanistic insights into the role of various oxidants in Au(I)/Au(III) oxidative couplings (Scheme 54) [124]. Oxidation of Au(I) complex with PhI(OAc)_2_ in the presence of *N*-methylindole cleanly gave the hetero-coupling product (**126**) in 80% yield; this transformation was also successful using electron-rich arenes 1,3,5- and 1,2,5-trimethoxybenzene. Interestingly, the same transformation using PhICl_2_ as the external oxidant was only applicable to *N*-methylindole as a substrate. Upon oxidation to Au(III) **127**, arene-auration can occur through two modes: (1) electrophilic aromatic substitution to give **128** or (2) concerted C-H activation via **129**, both of which converge to give intermediate **130**, which undergoes C–C bond-forming reductive elimination. The reactivity difference between PhI(OAc)_2_ and PhICl_2_ indicate that the basicity of the in-situ generated counterion may play a key role and, analogous to Sanford’s work in Pd(II)/PhI(OAc)_2_ C–H activation [2], the acetate group may assist in the key activation step (**129**). This would account for the diminished reactivity seen with PhICl_2_ in the case of substrates possessing less acidic C–H bonds such as 1,3,5- and 1,2,5-trimethoxybenzene, and suggest that PhI(OAc)_2_ may be superior to PhICl_2_ for Au(III)-mediated C–H activation.

**Scheme 55:**
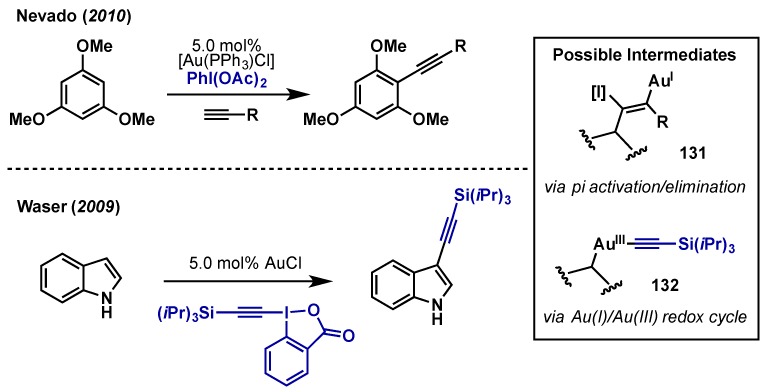
Oxidative alkynylation reactions by Nevado and Waser. Two potential pathways for alkynylation based upon Au(I) oxidation or Au(I) pi activation.

Au(III)-catalyzed arene alkynylations have been reported by both Nevado [125] and Waser [126], employing PhI(OAc)_2_ and an alkynyl benziodoxolone respectively (Scheme 55). The development of gold-mediated alkynylations of this type has been recently reviewed [127]. Nevado’s work used PhI(OAc)_2_ as an oxidant to couple electron-withdrawn alkynes and unactivated, electron-rich arenes; oxidants including PhIO, Selectfluor, and TBHP gave significantly lower yields. Waser utilized a slightly different approach wherein 1-[(triisopropylsilyl)ethynyl]-1λ^3^,2-benziodoxol-3(1*H*)-one (TIPS-EBX) served as both the oxidant and alkyne source, in the direct C3-alkynylation of indoles. Waser later extended this method to the alkynylation of other electron-rich heterocycles including thiophenes, anilines, and furans [128,129,130]. Although Au(I)/Au(III) redox couples are proposed in these cases via intermediates such as **132**, Au(I) carbophilic pi activation cannot dismissed as subsequent α- or β-elimination could provide the desired products via iodo-Au(I) intermediate **131**.

**Scheme 56:**
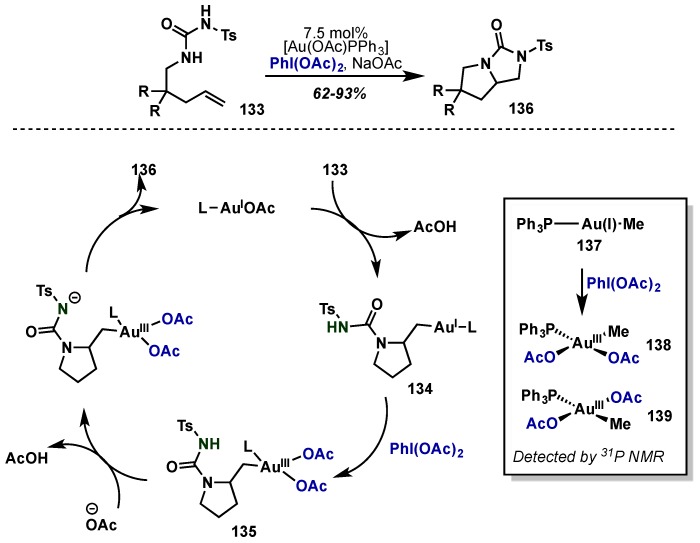
Au(I)/Au(III) catalytic cycle for intramolecular diamination of olefins.

In 2009, Muñiz and Iglesias took advantage of highly reactive Au(III) complexes to develop a gold-catalyzed alkene diamination reaction (Scheme 56), analogous to their previous report employing Pd(II)/Pd(IV) (see Section 2.2.4.1) [131]. Alkenes underwent intramolecular diamination with tosyl-protected ureas **133** under basic conditions using [Au(OAc)PPh_3_] to give bicyclic ureas (**136**) in high yield. Redox neutral anti-aminoauration gives Au(I) intermediate **134**, followed by irreversible oxidation by PhI(OAc)_2_ to Au(III) diacetate **135**. Following deprotonation, S_N_2-type intramolecular cyclization provides the desired cyclic urea **136** and regenerates the Au(I) catalyst. Although the proposed Au(III) intermediates were too reactive to be isolated, mechanistic studies using a PPh_3_–Au(I)–Me complex **137** gave an isomeric mixtures of Au(III) intermediates **138** and **139** upon oxidation with PhI(OAc)_2_, which were detectable by ^31^P-NMR.

**Scheme 57:**
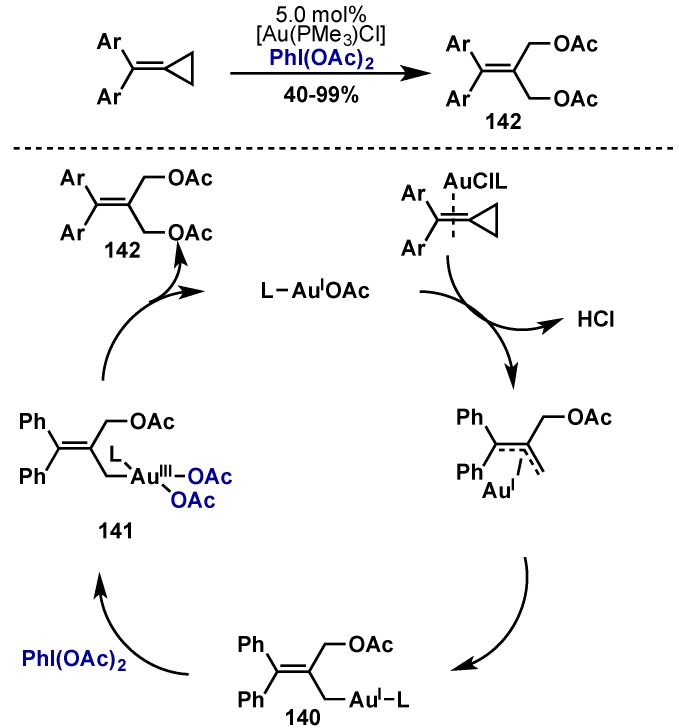
Au(I)/Au(III) catalyzed diacetoxylation of methylenecyclopropanes via C–C bond cleavage.

An interesting example of C–C bond cleavage was demonstrated by Shi and co-workers with Au(I)/Au(III) catalysis and methylenecyclopropanes (Scheme 57) [132]. Precomplexation of alkene to Au(I) leads to a redox neutral allylic rearrangement to give **140**, which is oxidized with PhI(OAc)_2_ to give Au(III) diacetate **141**. Reductive elimination from **141** would give the desired diacetate **142** with regeneration of a [Au(I)(PMe_3_)OAc] catalyst.

### 4.4. Conclusions

Although advancements have been made toward mechanistic understanding of Au(I)/Au(III)-mediated oxidative couplings, it is clear that synthetic applications of gold redox chemistry are still in their infancy. As methods development and mechanistic elucidation in this field continues, the choice of external oxidant play a crucial role as it affects both the stability of the reactive Au(III) intermediates and their subsequent reactivity in organic transformations. Thus far, PhICl_2_ has emerged as a leader for the isolation of Au(III) complexes, due the mild reaction conditions and stabilization imparted by the transfer of chloride ligands. Conversely, PhI(OAc)_2_ is more efficient in catalytic manifolds as the resultant complexes are more reactive and acetate ligands can assist in key C–H activation steps. The work of Dutton in the use of (poly)cationic λ^3^-iodanes has laid the groundwork for the exploration of highly tunable Au(III) complexes and this discovery could lead to new developments in the chemistry of cationic Au(III) intermediates.

## 5. Nickel

### 5.1. Introduction

**Scheme 58:**
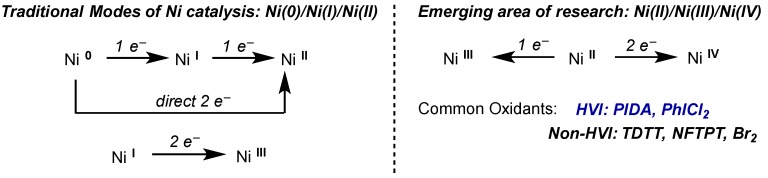
Traditional and emerging nickel oxidation states invoked in catalysis, and common oxidants reported in the literature.

Nickel is a group 10 metal, the first-row counterpart of palladium. Low oxidation state nickel redox couples, such as Ni(0)//Ni(I)/Ni(II), have seen significant applications in catalysis, including Suzuki, Negishi, and Kumada couplings, as well as recent advancements in radical mediated cross-coupling reactions [133]. A recent resurgence in nickel-catalyzed processes has been fueled not only by its greater sustainability and economic advantages, but also by its unique electronic properties that facilitate reactivity inaccessible to its palladium counterpart. Unlike palladium, which relies almost exclusively on two-electron redox cycles, nickel can undergo facile one- and two-electron redox events, providing access to Ni(0), Ni(I), Ni(II), Ni(III), and in rare examples, Ni(IV) oxidation states (Scheme 58). While these numerous pathways expand the scope of reactivity, they also make characterization and mechanistic investigations difficult due to the wide range of redox couples that could be invoked. Recent advancements in high oxidation state palladium chemistry has sparked a renewed investigation into the synthesis and characterization Ni(III) and Ni(IV) species, which could greatly expand the current scope of nickel-catalyzed reactions. In this section, we will highlight the key role that both ligand scaffold and oxidant can serve in the selection between either one- or two-electron pathways as well as the stability of the resultant high valent complexes. Hypervalent iodine oxidants have played a key role in the advancement of this field, analogous to their prominent role in the development of Pd(II)/Pd(IV) catalysis (see Section 2.1). In order to put modern approaches into context, we will begin with a brief history of isolated Ni(IV) complexes accessed via a variety of methods.

**Scheme 59:**
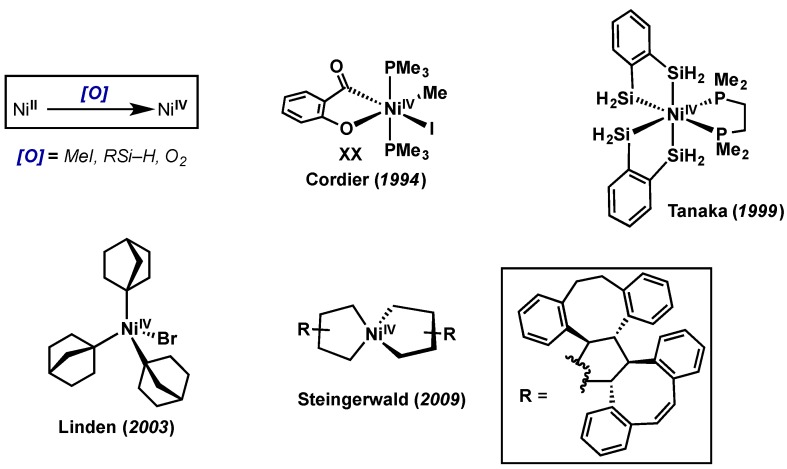
Examples of isolated organonickel (IV) complexes. Highly rigid ligand scaffolds and strong σ-donor ligands are common features that aid in stabilization of these species.

Reports on the isolation of Ni(IV) complexes date back to the mid 1970’s, and these species were even proposed as intermediates in some of the first Ni(0) catalyzed cross coupling reactions. The first diorganonickel (IV) complex was synthesized and isolated by Cordier in 1994 through oxidative addition of methyl iodide to a Ni(II) precursor with acylphenolato and trimethylphosphine ligands (Scheme 59) [134]. In this case, the rigid chelate ring contains a hard base and powerful σ-donating phosphine ligands that provide substantial stability to the high-oxidation nickel complex. In 1999, Tanaka et al reported the first silylnickel(IV) complex (Scheme 59) through a proposed oxidative addition of 1,2-disilylbenzene to a Ni(dmpe)_2_ and subsequent elimination of H_2_ [135]. Again, this complex was stabilized by strong σ-donor ligands and a rigid chelate similar to that of Cordier, and in both reports, X-ray crystallographic analysis confirmed an octahedral geometry. In 2003, Linden reported an isolable Ni(IV) complex containing three sigma bonded norbonyl ligands in a pseudo-tetrahedral geometry (Scheme 59) [136]. This species was accessed via oxidation of a tris(1-norbornyl)nickel(II) complex anion with O_2_ at −60 °C. Along with being strong σ-donor ligands, the steric bulk of the 1-norbonyl ligands provides shielding necessary for stabilization of the trialkylnickel(IV) species. Most recently, the Steigerwald group isolated a tetraalkyl aspirocyclononane Ni(IV) complex that is remarkably stable to oxygen and high temperatures (Scheme 59) [137]. The complex was isolated as an intermediate in a Ni(0) catalyzed strain release ring-opening polymerization, formed via the combination of two molecules of substrate into a dimeric bismetallocyclopentane nickel species. The remarkable stability of this complex is attributed to the high degree of steric shielding afforded by the large alkyl ligands. From these reports, common features emerge which stabilize high oxidation state nickel complexes: strong σ-donor ligands, rigid chelation, and steric shielding of the metal center. Additionally, these examples highlight the unusual stability of the nickel–alkyl bond, a feature not common in late transition metal organometallic complexes. Finally, a wide range of oxidants, varied in mechanism and strength, were utilized to access these systems, ranging from C–X oxidative addition to O_2_ oxidation at low temperature. While these studies provide valuable evidence for the viability of Ni(IV) species and the oxidants capable of accessing them, they are not readily translatable to catalytic manifolds.

Over the last 10 years, a renewed interest in this has focused on the subsequent reactivity and possible synthetic utility of Ni(IV) species, along with ligand scaffolds and oxidants that could be developed into viable catalytic systems. In this effort, hypervalent iodine reagents have emerged as promising candidates for both species isolation and catalysis.

### 5.2. Stoichiometric Studies

Continued efforts in the stoichiometric synthesis and characterization of Ni(IV) complexes have expanded to include their subsequent reactivity, particularly in C–C and C–X bond forming reductive eliminations. The most challenging element of this work has been species isolation and proof of either Ni(II)/Ni(III) or Ni(II)/Ni(IV) redox couples, as these intermediates are highly reactive and short lived.

**Scheme 60:**
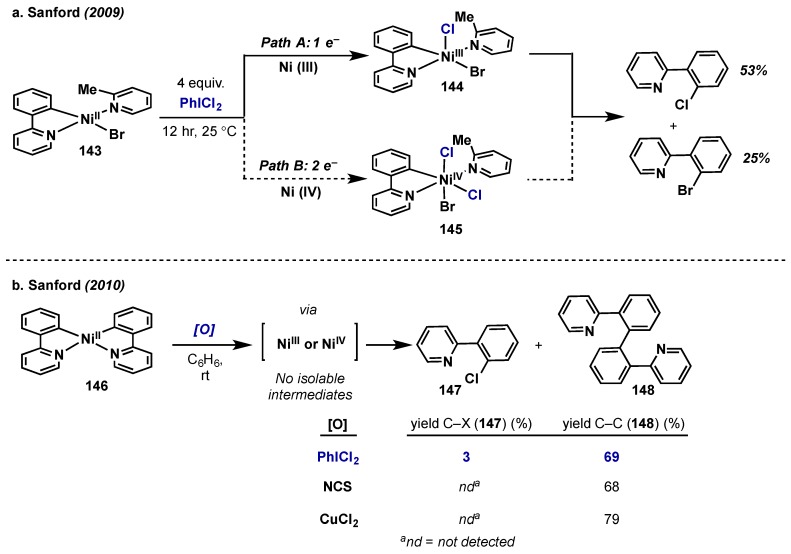
C–X versus C–C bond forming reductive elimination from high oxidation state nickel. (**a**) Competitive C–Br vs. C–Cl bond formation; (**b**) Competitive C–Cl vs. C–C bond formation.

The Sanford group has spearheaded these efforts, building on their extensive work in high oxidation state palladium chemistry. They have explored the viability of both Ni(III) and Ni(IV) manifolds as inexpensive analogues to Pd(IV) in C–X reductive eliminations. A 2010 report studied the oxidation of complex **143** with an excess of PhICl_2_, which yielded an approximately 2:1 mixture of C–Cl and C-Br reductive elimination products (Scheme 60a) [138]. This represented the first example of C–X bond forming reductive elimination from nickel. They propose the likely intermediacy of a Ni(III) species (**144**), however note that two-electron oxidation to Ni(IV) (**145**) cannot be ruled out due to an inability to isolate the reactive intermediates. It is also noteworthy that both the chlorine ligands introduced upon oxidation, and the bromine ligand present in complexes **144** and **145**, were available for C–X reductive elimination.

A subsequent study by Sanford examined the oxidation of a Ni(PhPy)_2_ complex (**146**), which can then undergo competitive C–X or C–C reductive elimination (Scheme 60b) [139]. The analogous palladium complex had previously been shown to undergo preferential C–X reductive elimination when exposed to a variety of oxidants, including hypervalent iodine reagents (see Section 2.2.3.1), and thus this study provided an excellent comparison of the reactivity of these two metals. It was shown that upon oxidation with PhICl_2_, complex **146** gave only trace C–Cl bond formation (3%, **147**) and the major product **148** was that of C–C reductive elimination. Interestingly, oxidation of **146** with other Cl^+^ sources, NCS or CuCl_2_, provided no observable C–Cl products, showing a slight but significant divergence in reactivity of the hypervalent iodine oxidant. Again, in this study, no high oxidation state intermediates could be isolated or observed in situ, although the divergent reactivity from that of palladium may suggest a difference in mechanism.

**Scheme 61:**
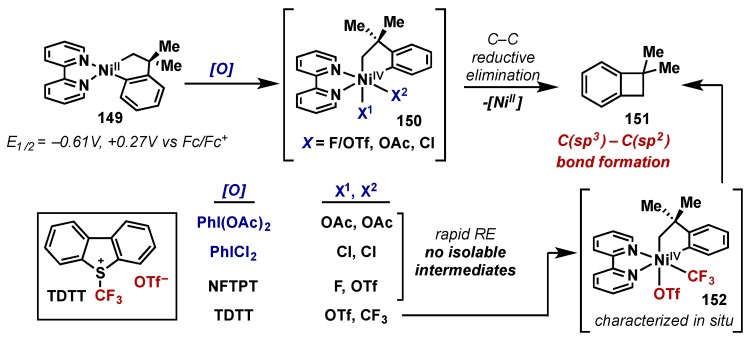
Oxidation and reductive elimination from a Ni(IV) intermediate using hypervalent iodine, F^+^, and CF_3_^+^ sources. Rapid and selective C–C bond formation was observed in the case of all hypervalent iodine oxidants.

Recently, in a high-profile report, the same group succeeded in the isolation and characterization of a Ni(IV) intermediate upon oxidation of a diorganonickel(II) complex **149** (Scheme 61) [140]. At the outset of their study, key insights into the stability of **149** to oxidation were obtained via cyclic voltammetry. Observation of two quasi-reversible oxidation peaks at −0.61 V and +0.27 vs. Fc/Fc^+^, representative of Ni(II)/Ni(III) and Ni(III)/Ni(IV) redox couples, is noteworthy as these potentials are relatively low and hints that catalytic cycles with interchange between these redox states could be plausible. Initially, oxidation of **149** with hypervalent iodine reagents PhI(OAc)_2_, PhICl_2_, or F^+^ source NFTPT resulted in rapid C(sp^3^)–C(sp^2^) reductive elimination to give cyclobutane **151**, not allowing for study of any intermediates. However, use of TDTT resulted in formation of a stable Ni(IV)–CF_3_ intermediate **152** that was detectable by ^1^H- and ^19^F-NMR, before giving rise to the same cyclobutane product **151**. Further evidence for the intermediacy of a Ni(IV) species was provided through X-ray crystallographic characterization of a complex possessing a tridentate tris(2-pyridyl) methane ligand, again upon oxidation with TDTT (not shown). Although products of oxidation with hypervalent iodine reagents were not directly observed in this case, the analogous reactivity of complex **150** to that of hypervalent iodine-mediated oxidation provides strong evidence that those reactions are also proceeding via a Ni(II)/Ni(IV) redox couple. This study provides clear evidence for the accessibility of Ni(II)/Ni(IV) catalysis manifolds and supports further efforts for developing reactions that invoke this pathway. It also shows that the development of novel C–X bond-forming reactions, analogous to those with Pd(II)/Pd(IV), will likely require ligand scaffolds that are not capable of undergoing competitive C–C reductive elimination.

**Scheme 62:**
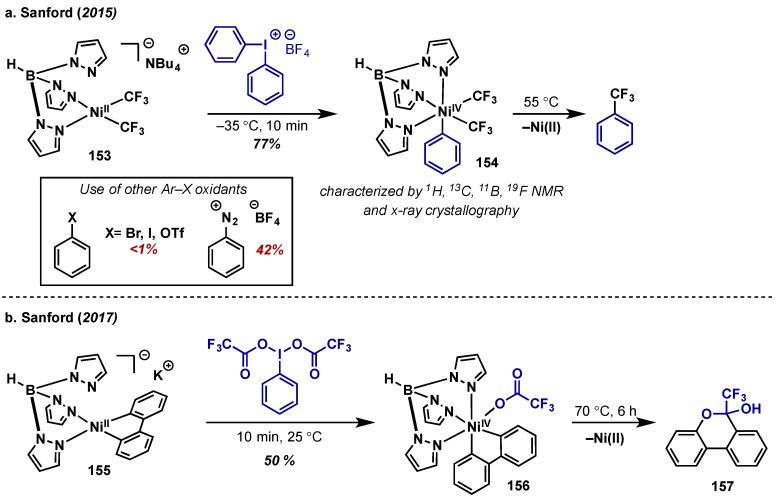
Use of tripyrazoylborate (Tp) ligand in the synthesis and characterization of Ni(IV) species via oxidation with hypervalent iodine reagents. (**a**) Use of diaryliodonium salts; (**b**) Use of PhI(OTFA)_2_.

Sanford’s group has recently utilized tripyrazoylborate (Tp) as a supporting ligand to isolate Ni(IV) complex **153** and examine its competency in C(sp^2^)–CF_3_ reductive eliminations (Scheme 62a) [141]. Oxidation was examined with a variety of Ar–X species and it was found that only aryldiazonium salts and diaryliodonium salts were competent oxidants, with diaryliodonium salts giving superior yields of complex **154**. This represents the first evidence of the accessibility of the Ni(II)/Ni(IV) manifold under mild conditions with diaryliodonium salts. **154** was further shown to undergo clean C–CF_3_ bond formation upon heating and results suggest a two-electron reductive elimination pathway. Sanford also recently reported the oxidation of a TpNi(II)biphenyl complex **155** and its subsequent reactivity in C–O bond reductive elimination (Scheme 62b) [142]. Oxidation with PhI(OTFA)_2_ for just 10 min at 25 °C cleanly produced **156** in 50% isolated yield. In line with their previous studies, intramolecular C(sp^2^)–O coupling from **156** was slow, however **157** could be detected upon heating, the result of C–O bond-forming reductive elimination followed by cyclization and hemiketalization. It was also found that the –OTFA ligand could under rapid displacement with a nucleophilic source of –CF_3_, which would then undergo slow C–CF_3_ reductive elimination (not shown). Taken together, these two reports show the relatively facile access of the Ni(II)/Ni(IV) manifold with hypervalent iodine reagents and lend strong support for the development of diverse catalytic reactions via this pathway. Furthermore, the tripyrazoylborate ligand is emerging as an excellent choice for stabilization and isolation of high oxidation state nickel complexes.

**Scheme 63:**
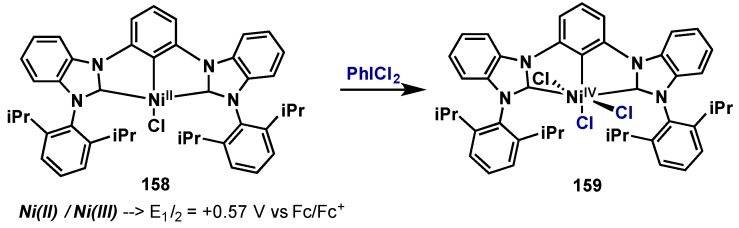
Two electron oxidation of Ni(II) monoanionic bis(carbene) pincer complex with PhICl_2_ and characterization of stable Ni(IV) intermediate **159**.

Taking advantage of the excellent σ-donor ability of *N*-heterocyclic carbenes (NHCs), Alison Fout’s group reported the isolation of Ni(IV) complex **159** and its reactivity has an electrophilic halogen surrogate (Scheme 63) [143]. Initial characterization of Ni(II) species **158** via cyclic voltammetry revealed only a single reversible oxidation wave at +0.57 V vs. Fc/Fc^+^, which was attributed to the Ni(II)/Ni(III) couple. Thus, it was somewhat surprising that treatment of **158** with common outer-sphere one-electron oxidants such as Ag^+^, Ph_3_C^+^, or Fc^+^, was unsuccessful. However, treatment of **158** with PhICl_2_ resulted in clean two-electron oxidation to give **159**, which was characterized by X-ray crystallography. This result was the first definitive evidence that hypervalent iodine reagents were competent oxidants in the Ni(II)/Ni(IV) redox couple. Also notable is the significantly higher oxidation potential of **158** relative to that of **149** (see Scheme 61) and yet PhICl_2_ was still a competent oxidant. In the continued development of this field, it will likely be important to continue to obtain electrochemical measurements of both hypervalent iodine reagents and metal complexes, which could then be used as a predictive tool for the success of the subsequent oxidation.

### 5.3. Synthetic Applications

Catalytic cycles, and thus also synthetic applications, invoking high oxidation state nickel intermediates remain quite rare. However, building on the body of work in stoichiometric complex synthesis, such reports are beginning to emerge, but the oxidation states in play are difficult to discern. Below we outline recent catalytic reports invoking both Ni(III) and Ni(IV) intermediates via oxidation with hypervalent iodine reagents as well as synthetically relevant applications involving stoichiometric complexes.

**Scheme 64:**
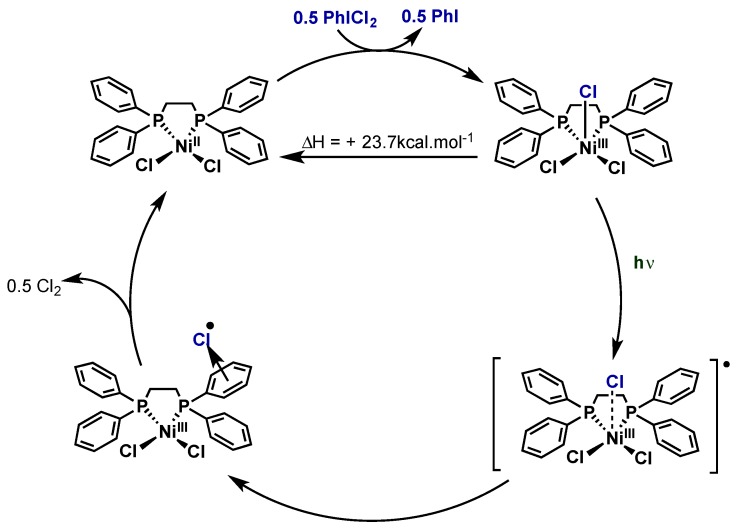
Photoelimination of Cl_2_ via single electron Ni(II)/Ni(III) cycle mediated by PhICl_2_.

The Nocera group reported the catalytic photoelimination of Cl_2_ from a Ni(III) intermediate, accessed via one-electron oxidation of a Ni(II) species with PhICl_2_ (Scheme 64) [144]. In this case, careful control of the equivalents of PhICl_2_ allows for selective transfer of a single chlorine atom. It is also possible that the ligand scaffold does not lower the oxidation potential far enough to make a subsequent Ni(III)/Ni(IV) oxidation accessible in this case, however electrochemical measurements on this complex were not provided.

**Scheme 65:**
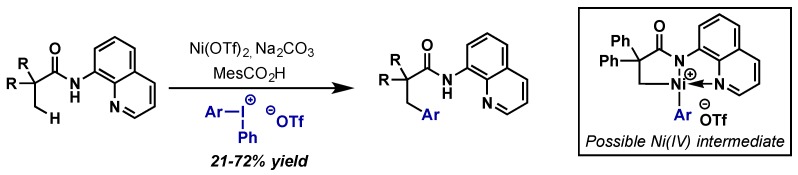
C(sp^3^)–H arylation with diaryliodonium salts through a proposed Ni(II)/Ni(IV) catalytic cycle.

Chatani’s group invoked a Ni(II)/Ni(IV) catalytic cycle in their development of a directed C(sp^3^)–H arylation with diaryliodonium salts (Scheme 65) [145]. A variety of diaryliodonium salts bearing different counter ions such were examined, however high yields were only obtained in the case of triflate, presumably due to the necessary regeneration of the Ni(OTf)_2_ catalyst. Although no intermediates were isolated, radical trap experiments with TEMPO gave no TEMPO-adducts, providing at least preliminary evidence against one-electron pathways.

**Scheme 66:**
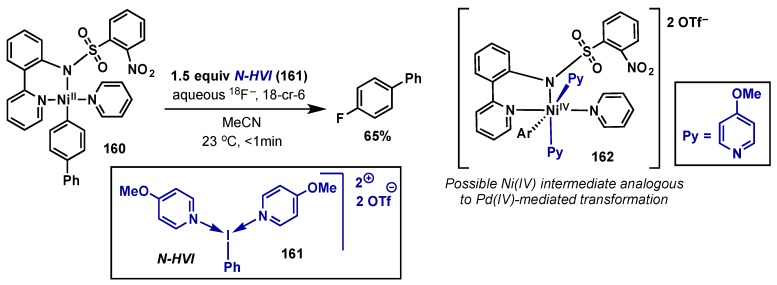
Ritter’s nickel-mediated radiofluorination via oxidation of Ni(II) with (poly)cationic λ^3^-iodane **161**, possessing neutral heterocycle ligands.

Continuing their work in oxidative fluorination chemistry (for palladium analogue see Section 2.2.3.1), the Ritter group reported the development of a one-step oxidative radiofluorination of arenes employing Ni(II) complex **160** with aqueous ^18^F, and poly(cationic) *N*-ligated λ^3^-iodane **161** as the oxidant (Scheme 66) [146]. This one-step oxidation/C–F bond formation proceeds via the two-electron oxidation of **160** by cationic hypervalent iodine **161**, which undergoes ligand exchange with nucleophilic fluoride and subsequent reductive elimination. The use of (poly)cationic λ^3^-iodane **161** is critical in this case as the desired ^18^F reductive elimination could easily be outcompeted by the –X ligands transferred to the metal center with traditional hypervalent iodine complexes (i.e., –Cl, –OAc, –OTFA) [40]. Instead complex **161** transfers two “innocent” heterocyclic ligands, allowing for the challenging C–F reductive elimination to proceed in high yield. Even though the detailed mechanistic studies were not reported, the formation of Ni(IV) intermediate **162** is reasonable by analogy to their prior reports, as well as work by Dutton employing (poly)cationic λ^3^-iodanes as two-electron oxidants in the generation of Pd(IV) and Pt(IV) species (see Section 3.1). This work shows the promise of the relatively unexplored class of (poly)cationic λ^3^-iodanes as powerful oxidants that allow for challenging reductive eliminations that would be precluded with the use of more common oxidants.

### 5.4. Conclusions

High valent nickel catalysis, specifically via the Ni(II)/Ni(IV) redox couple is an emerging area of research that has the potential to offer novel and powerful reactivity. The continued growth and maturation of this area will rely on continued efforts to understand the role that ligand scaffold and oxidant can play in the oxidation states accessible, as well as studies aimed at the isolation and characterization of these high oxidation state complexes. Furthermore, a more detailed mechanistic understanding of reported synthetic methods will educate further reaction development.

## 6. Copper

### 6.1. Introduction

**Scheme 67:**
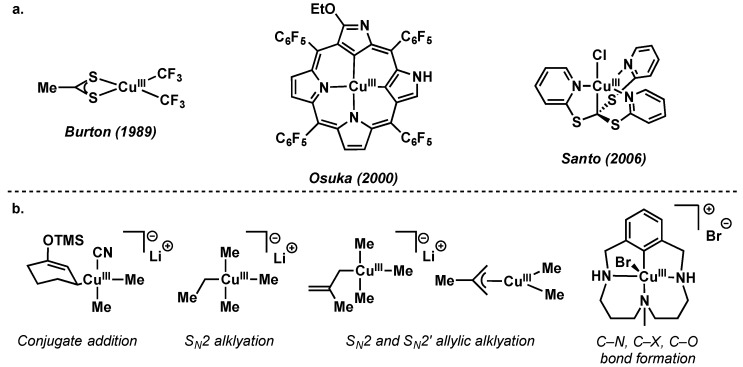
(**a**) Examples of isolable Cu(III) complexes; (**b**) Observed Cu(III) species by rapid-injection (RI) NMR spectroscopy.

As recently as the year 2000, reports of isolable high-valent organometallic Cu(III) species were extremely rare (Scheme 67a) [147,148,149] and little was known about the potential role of Cu(I)/Cu(III) cycles in catalysis. However, significant progress has been made in this area over the last 15 years and modern analytical techniques such as rapid-injection NMR (RI-NMR) have provided clear evidence of Cu(III) intermediates in carbon-carbon, carbon-halogen, and carbon-nitrogen bond forming reactions (Scheme 67b) [1]. Applications of hypervalent iodine reagents in this area are scarce however one could imagine that this will be a fruitful area of research in the coming years. In this section recent examples of proposed Cu(I)/Cu(III) redox cycles involving hypervalent iodine reagents will be presented, though the underlying mechanisms of these transformations are still the subject of debate in the literature.

### 6.2. Synthetic Applications

**Scheme 68:**
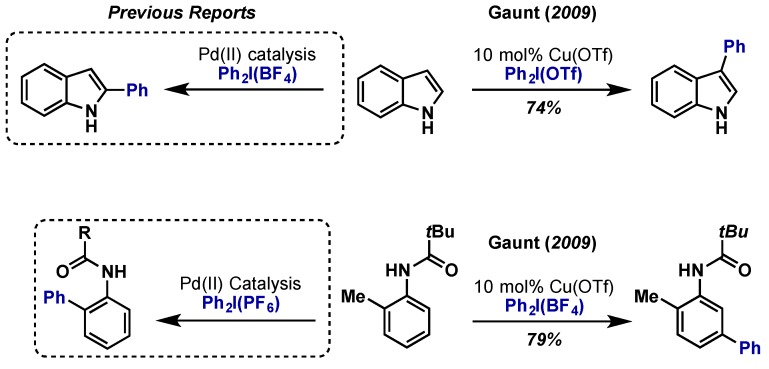
Copper-catalyzed oxidative arylation reactions with diaryliodonium salts show complimentary regioselectivity to analogous palladium-catalyzed systems.

**Scheme 69:**
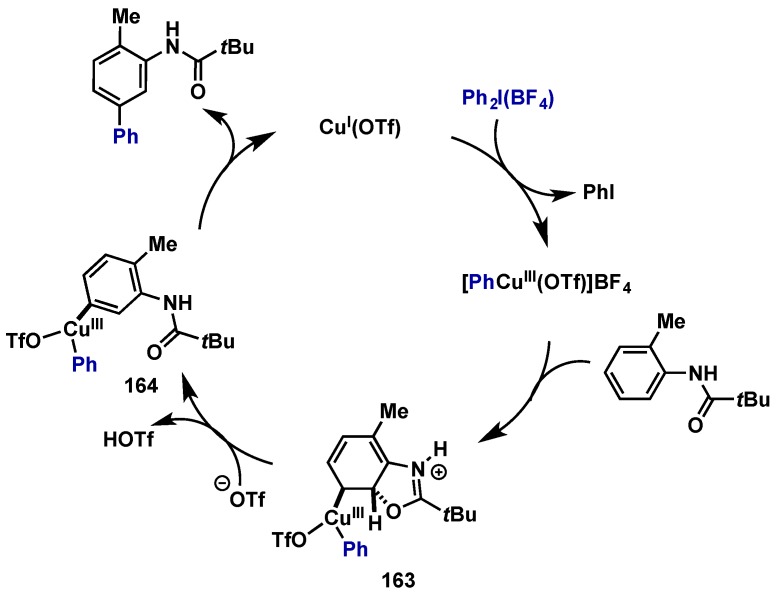
Proposed Cu(I)/Cu(III) catalytic cycle for C–H functionalization of benzene derivatives.

Gaunt and co-workers have reported landmark examples of Cu(I)/Cu(III) catalysis through C–H arylation of indoles and benzene derivatives with diaryliodonium salts (Scheme 68) [150,151]. Remarkably, these arylations proceeded with extremely high *C3*- and *meta*-selectivity respectively, complimentary to the analogous Pd(II)-catalyzed transformations which give exclusively products arising from directed C–H activation. Shown in the context of arene functionalization (Scheme 69), the authors propose a catalytic cycle involving Ph_2_IBF_4_-mediated oxidation of Cu(I) to a highly electrophilic Cu(III) complex **163**, accompanied by aryl transfer. This species then undergoes *meta*-selective Friedel-Crafts-type metalation, facilitated by the pendant amide, followed by rearomatization to give Cu(III) complex **164**. C–C bond forming reductive elimination would provide the desired arylated product and regenerate Cu(I)OTf. While this mechanism explains the observed selectivity, the intermediacy of Cu(III) in these processes has not been confirmed experimentally. The authors note that the addition of a radical inhibitor, 1,1-diphenylethylene, does not inhibit product formation, suggesting a radical mechanism is unlikely. In contrast, Buchwald has shown that Cu(I)-catalyzed processes involving Togni’s reagent proceed via one-electron radical cascades [152].

### 6.3. Conclusions

The past 15 years have provided critical experimental evidence for the intermediacy of Cu(III) in a variety of catalytic transformations. Hypervalent iodine reagents have been applied in seminal reports by Gaunt using diaryliodonium salts in C–H arylation, however experimental evidence supporting a Cu(I)/Cu(III) pathway is still lacking. Therefore, while the potential of hypervalent iodine reagents in high-valent copper catalysis is immense, the continued development of this area will require a more detailed mechanistic understanding of the pathways involved.

## 7. Miscellaneous High Valent Metal Complexes

In addition to the applications discussed thus far, hypervalent iodine reagents have been utilized as oxidants for a wide range of metals less prevalent in traditional catalysis. These include oxidations of Mo, W, V, Ce, Ir, Fe and Rh, with a large focus on high valent complex isolation but a few recent examples also include catalytic reaction development. In this area, PhICl_2_ and PhI(OAc)_2_ have been the most widely applied due to their well-studied reactivity and their inherent transfer of chloride and acetate ligands capable of stabilizing high oxidation state species.

### 7.1. Complex Synthesis and Isolation

**Scheme 70:**
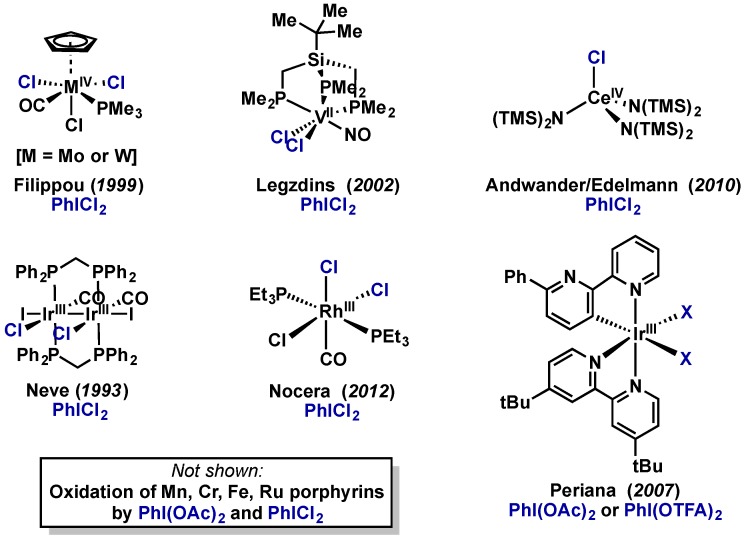
Examples of high oxidation state transition metal complexes accessed by oxidation with PhI(OAc)_2_ and PhICl_2_.

Scheme 70 summarizes the diverse metal complexes that have been accessed via hypervalent iodine oxidants. These complexes vary in their oxidation states, geometries, ligand scaffolds, and were accessed through both one- and two-electron oxidation pathways. Filippou utilized PhICl_2_ in an oxidative decarbonylation approach to accessing Mo(IV) and W(IV) complexes en route to studying trichlorogermyl compounds [153]. Legzdins synthesized a V(II) nitrosyl complex using PhICl_2_ in order to study the nitrosyl ligand effects on early transition metals [154]. In an effort to improve the syntheses of Ce(IV) amides, Andwander and Edelmann synthesized a Ce(IV) complex with use of PhICl_2_ as a one-electron oxidant [155]. Neve used PhICl_2_ to study the structure and properties of an Ir(III) dimer [156]. A report from Nocera synthesized Rh(III) complexes with PhICl_2_ as part of a study to understand the role of Rh(III) hydride complexes in the reduction of oxygen to H_2_O in reactions with HCl [157]. Periana demonstrated functionalization of an Ir(V) complex with both PhI(OAc)_2_ and PhI(OTFA)_2_ in order study oxy-functionalization reactions from high oxidation state iridium [158]. Although not shown in Scheme 70, PhI(OAc)_2_ and PhICl_2_ also have rich histories as co-oxidants in the study of metalloporphyrins [159,160,161].

### 7.2. Catalytic Applications

**Scheme 71:**
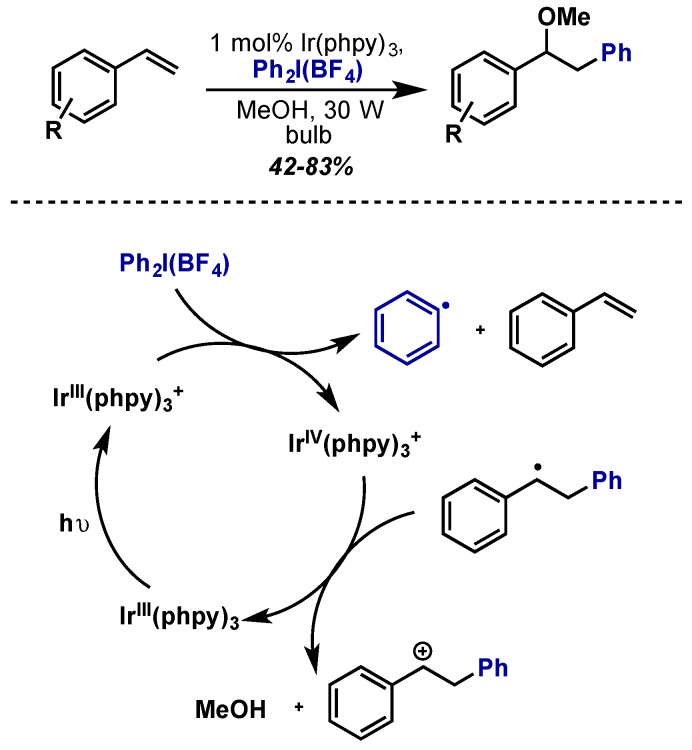
Ir(III)(phpy)_3_ catalyzed photoredox oxyarylation of styrene derivatives.

Although one-electron manifolds are not prototypical of hypervalent iodine reagents, diaryliodonium salts have been reported to serve as one-electron oxidants in both iron and iridium catalyzed transformations. Photoredox catalysis using Ir(III)(phpy)_3_, with Ph_2_I(BF_4_) as both the external oxidant and phenyl source, was successfully applied to the methoxyphenylation of styrene derivatives (Scheme 71) [162]. It is proposed that Ph_2_I(BF_4_) transfers a phenyl radical to styrene with in situ oxidation of Ir(III)(phpy)_3_^+^ to Ir(IV)(phpy)_3_^+^. Further oxidation to a benzylic cation followed by trapping with methanol gives the difunctionalized product.

**Scheme 72:**
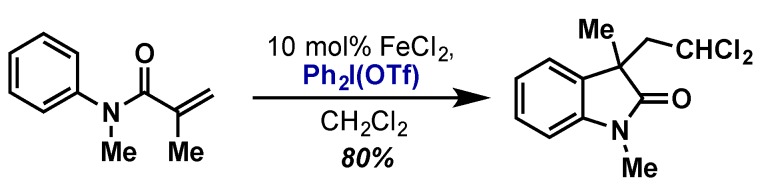
FeCl_2_ catalyzed carbochloromethylation of activated alkenes.

Loh has shown that Ph_2_I(OTf) can act as a one-oxidant in an Fe(II)/Fe(III) catalyzed intramolecular radical cyclization cascade (Scheme 72) [163]. It is proposed that Ph_2_I(OTf) produces a phenyl radical upon oxidation of Fe(II) to Fe(III) with subsequent hydrogen abstraction from dichloromethane. Although these one-electron pathways remain rare, these reports show promise for the further development of hypervalent iodine reagents in these manifolds.

### 7.3. Conclusions

The versatility of hypervalent iodine reagents is evident in the wide range of metal centers that they are capable of oxidizing. Their application in the isolation and characterization of late transition metal complexes provides critical insights into the further development and application of these species. Furthermore, their ability to facilitate one-electron pathways has enabled their extension into metal catalyzed photoredox and radical cascade reactions.

## 8. Conclusions

High valent metal catalysis is still an emerging field that continues to be rich with opportunities for novel and creative reaction development. Hypervalent iodine reagents have emerged as versatile oxidants in this area, providing versatile reactivity, heteroatom ligands, and mild reaction conditions. These reagents are also environmentally benign, non-toxic, and relatively inexpensive compared to other inorganic oxidants. Despite their broad utility, there remain limitations in their application; they produce stoichiometric organic byproducts, there is a limited scope of heteroatoms ligands available, and their use in facilitating challenging reductive eliminations (i.e., –CF_3_, –F) is limited. Future developments in more diverse hypervalent iodine scaffolds, particularly in those that could act as “innocent” oxidants, or those that could be readily recycled/regenerated, has the potential to greatly expand the utility of this already powerful reaction manifold.

## References

[1] Hickman A.J., Sanford M.S. (2012). High-valent organometallic copper and palladium in catalysis. Nature.

[2] Lyons T.W., Sanford M.S. (2010). Palladium-Catalyzed Ligand-Directed C–H Functionalization Reactions. Chem. Rev..

[3] Canty A.J. (1992). Development of organopalladium(IV) chemistry: Fundamental aspects and systems for studies of mechanism in organometallic chemistry and catalysis. Acc. Chem. Res..

[4] Xu L.-M., Li B.-J., Yang Z., Shi Z.-J. (2010). Organopalladium(IV) chemistry. Chem. Soc. Rev..

[5] Sehnal P., Taylor R.J.K., Fairlamb I.J.S. (2010). Emergence of Palladium(IV) Chemistry in Synthesis and Catalysis. Chem. Rev..

[6] Dick A.R., Sanford M.S. (2006). Transition metal catalyzed oxidative functionalization of carbon–hydrogen bonds. Tetrahedron.

[7] Topczewski J.J., Sanford M.S. (2014). Carbon–hydrogen (C–H) bond activation at Pd^IV^: A frontier in C–H functionalization catalysis. Chem. Sci..

[8] Racowski J.M., Sanford M.S. (2011). Carbon–Heteroatom Bond-Forming Reductive Elimination from Palladium(IV) Complexes. Higher Oxidation State Organopalladium and Platinum Chemistry.

[9] Byers P.K., Canty A.J., Skelton B.W., White A.H. (1986). The oxidative addition of lodomethane to [PdMe_2_(bpy)] and the X-ray structure of the organopalladium(IV) product fac-[PdMe_3_(bpy)_1_](bpy = 2,2′-bipyridyl). J. Chem. Soc. Chem. Commun..

[10] Yoneyama T., Crabtree R.H. (1996). Pd(II) catalyzed acetoxylation of arenes with iodosyl acetate. J. Mol. Catal. A Chem..

[11] Stock L.M., Tse K.T., Vorvick L.J., Walstrum S.A. (1981). Palladium(II) acetate catalyzed aromatic substitution reaction. J. Org. Chem..

[12] Dick A.R., Hull K.L., Sanford M.S. (2004). A Highly Selective Catalytic Method for the Oxidative Functionalization of C–H Bonds. J. Am. Chem. Soc..

[13] Kalyani D., Sanford M.S. (2005). Regioselectivity in Palladium-Catalyzed C–H Activation/Oxygenation Reactions. Org. Lett..

[14] Kalberer E.W., Whitfield S.R., Sanford M.S. (2006). Application of recyclable, polymer-immobilized iodine(III) oxidants in catalytic C–H bond functionalization. J. Mol. Catal. A Chem..

[15] Desai L.V., Hull K.L., Sanford M.S. (2004). Palladium-Catalyzed Oxygenation of Unactivated sp^3^ C–H Bonds. J. Am. Chem. Soc..

[16] Neufeldt S.R., Sanford M.S. (2010). *O*-acetyl oximes as transformable directing groups for Pd-catalyzed C-H bond functionalization. Org. Lett..

[17] Wang D.-H., Hao X.-S., Wu D.-F., Yu J.-Q. (2006). Palladium-catalyzed oxidation of Boc-protected *N*-methylamines with IOAc as the oxidant: A Boc-directed sp^3^ C-H bond activation. Org. Lett..

[18] Cook A.K., Sanford M.S. (2015). Mechanism of the palladium-catalyzed arene C–H acetoxylation: A comparison of catalysts and ligand effects. J. Am. Chem. Soc..

[19] Dick A.R., Kampf J.W., Sanford M.S. (2005). Unusually Stable Palladium(IV) Complexes: Detailed Mechanistic Investigation of C–O Bond-Forming Reductive Elimination. J. Am. Chem. Soc..

[20] Racowski J.M., Dick A.R., Sanford M.S. (2009). Detailed study of C–O and C–C bond-forming reductive elimination from stable C2N2O2-ligated palladium(IV) complexes. J. Am. Chem. Soc..

[21] Cheng X.-F., Li Y., Su Y.-M., Yin F., Wang J.-Y., Sheng J., Vora H.U., Wang X.-S., Yu J.-Q. (2013). Pd(II)-catalyzed enantioselective C-H activation/C-O bond formation: Synthesis of chiral benzofuranones. J. Am. Chem. Soc..

[22] Emmert M.H., Cook A.K., Xie Y.J., Sanford M.S. (2011). Remarkably high reactivity of Pd(OAc)_2_/pyridine catalysts: Nondirected C-H oxygenation of arenes. Angew. Chem. Int. Ed..

[23] Zhang S.-Y., He G., Zhao Y., Wright K., Nack W.A., Chen G. (2012). Efficient alkyl ether synthesis via palladium-catalyzed, picolinamide-directed alkoxylation of unactivated C(sp^3^)–H and C(sp^2^)–H bonds at remote positions. J. Am. Chem. Soc..

[24] Shan G., Yang X., Zong Y., Rao Y. (2013). An efficient palladium-catalyzed C-H alkoxylation of unactivated methylene and methyl groups with cyclic hypervalent iodine (i(3+)) oxidants. Angew. Chem. Int. Ed..

[25] Liu G., Stahl S.S. (2006). Highly regioselective Pd-catalyzed intermolecular aminoacetoxylation of alkenes and evidence for *cis*-aminopalladation and S_N_2 C–O bond formation. J. Am. Chem. Soc..

[26] Desai L.V., Sanford M.S. (2007). Construction of tetrahydrofurans by PdII/PdIV-catalyzed aminooxygenation of alkenes. Angew. Chem. Int. Ed. Engl..

[27] Li Y., Song D., Dong V.M. (2008). Palladium-Catalyzed Olefin Dioxygenation. J. Am. Chem. Soc..

[28] Neufeldt S.R., Sanford M.S. (2013). Asymmetric chiral ligand-directed alkene dioxygenation. Org. Lett..

[29] Pilarski L.T., Selander N., Böse D., Szabó K.J. (2009). Catalytic allylic C-H acetoxylation and benzoyloxylation via suggested (eta(3)-allyl)palladium(IV) intermediates. Org. Lett..

[30] Alam R., Pilarski L.T., Pershagen E., Szabó K.J. (2012). Stereoselective intermolecular allylic C–H trifluoroacetoxylation of functionalized alkenes. J. Am. Chem. Soc..

[31] Bigi M.A., White M.C. (2013). Terminal Olefins to Linear α,β-Unsaturated Ketones: Pd(II)/Hypervalent Iodine Co-catalyzed Wacker Oxidation—Dehydrogenation. J. Am. Chem. Soc..

[32] Giri R., Chen X., Hao X.-S., Li J.-J., Liang J., Fan Z.-P., Yu J.-Q. (2005). Catalytic and stereoselective iodination of prochiral C–H bonds. Tetrahedron Asymmetry.

[33] Giri R., Chen X., Yu J.-Q. (2005). Palladium-catalyzed asymmetric iodination of unactivated C-H bonds under mild conditions. Angew. Chem. Int. Ed. Engl..

[34] Mei T.-S., Giri R., Maugel N., Yu J.-Q. (2008). Pd(II)-catalyzed monoselective ortho halogenation of C-H bonds assisted by counter cations: A complementary method to directed ortho lithiation. Angew. Chem. Int. Ed..

[35] McCall A.S., Wang H., Desper J.M., Kraft S. (2011). Bis-N-heterocyclic carbene Palladium(IV) tetrachloride complexes: Synthesis, reactivity, and mechanisms of direct chlorinations and oxidations of organic substrates. J. Am. Chem. Soc..

[36] Lee E., Kamlet A.S., Powers D.C., Neumann C.N., Boursalian G.B., Furuya T., Choi D.C., Hooker J.M., Ritter T. (2011). A fluoride-derived electrophilic late-stage fluorination reagent for PET imaging. Science.

[37] Furuya T., Kaiser H.M., Ritter T. (2008). Palladium-mediated fluorination of arylboronic acids. Angew. Chem. Int. Ed..

[38] Hull K.L., Anani W.Q., Sanford M.S. (2006). Palladium-catalyzed fluorination of carbon-hydrogen bonds. J. Am. Chem. Soc..

[39] Wang X., Mei T.-S., Yu J.-Q. (2009). Versatile Pd(OTf)_2_·2H_2_O-catalyzed ortho-fluorination using NMP as a promoter. J. Am. Chem. Soc..

[40] McMurtrey K.B., Racowski J.M., Sanford M.S. (2012). Pd-Catalyzed C–H Fluorination with Nucleophilic Fluoride. Org. Lett..

[41] Selander N., Willy B., Szabó K.J. (2010). Selective C–H borylation of alkenes by palladium pincer complex catalyzed oxidative functionalization. Angew. Chem. Int. Ed..

[42] Muñiz K., Martínez C., Iglesias A. (2016). The Quest for Palladium-Catalysed Alkyl-Nitrogen Bond Formation. Chem. Rec..

[43] Streuff J., Hövelmann C.H., Nieger M., Muñiz K. (2005). Palladium(II)-catalyzed intramolecular diamination of unfunctionalized alkenes. J. Am. Chem. Soc..

[44] Muñiz K., Hövelmann C.H., Streuff J. (2008). Oxidative diamination of alkenes with ureas as nitrogen sources: Mechanistic pathways in the presence of a high oxidation state palladium catalyst. J. Am. Chem. Soc..

[45] Iglesias A., Muñiz K. (2012). Studies on Alkyl-Nitrogen Bond Formation via Reductive Elimination from Monomeric Palladium Complexes in High Oxidation State. Helv. Chim. Acta.

[46] Hövelmann C.H., Streuff J., Brelot L., Muñiz K. (2008). Direct synthesis of bicyclic guanidines through unprecedented Palladium(II) catalysed diamination with copper chloride as oxidant. Chem. Commun. (Camb.).

[47] Welbes L.L., Lyons T.W., Cychosz K.A., Sanford M.S. (2007). Synthesis of cyclopropanes via Pd(II/IV)-catalyzed reactions of enynes. J. Am. Chem. Soc..

[48] Muñiz K. (2007). Advancing palladium-catalyzed C-N bond formation: Bisindoline construction from successive amide transfer to internal alkenes. J. Am. Chem. Soc..

[49] Iglesias A., Pérez E.G., Muñiz K. (2010). An Intermolecular Palladium-Catalyzed Diamination of Unactivated Alkenes. Angew. Chem. Int. Ed..

[50] Muñiz K., Kirsch J., Chávez P. (2011). Intermolecular Regioselective 1,2-Diamination of Allylic Ethers. Adv. Synth. Catal..

[51] Martínez C., Muñiz K. (2012). Palladium-catalyzed vicinal difunctionalization of internal alkenes: Diastereoselective synthesis of diamines. Angew. Chem. Int. Ed..

[52] Ingalls E.L., Sibbald P.A., Kaminsky W., Michael F.E. (2013). Enantioselective palladium-catalyzed diamination of alkenes using *N*-fluorobenzenesulfonimide. J. Am. Chem. Soc..

[53] Sibbald P.A., Michael F.E. (2009). Palladium-catalyzed diamination of unactivated alkenes using *N*-fluorobenzenesulfonimide as source of electrophilic nitrogen. Org. Lett..

[54] Dick A.R., Remy M.S., Kampf J.W., Sanford M.S. (2007). Carbon–Nitrogen Bond-Forming Reactions of Palladacycles with Hypervalent Iodine Reagents. Organometallics.

[55] Thu H.-Y., Yu W.-Y., Che C.-M. (2006). Intermolecular amidation of unactivated sp^2^ and sp^3^ C-H bonds via palladium-catalyzed cascade C–H activation/nitrene insertion. J. Am. Chem. Soc..

[56] Nadres E.T., Daugulis O. (2012). Heterocycle synthesis via direct C–H/N–H coupling. J. Am. Chem. Soc..

[57] He G., Zhao Y., Zhang S., Lu C., Chen G. (2012). Highly efficient syntheses of azetidines, pyrrolidines, and indolines via palladium catalyzed intramolecular amination of C(sp^3^)–H and C(sp^2^)–H bonds at γ and δ positions. J. Am. Chem. Soc..

[58] Iglesias A., Alvarez R., de Lera A.R., Muñiz K. (2012). Palladium-catalyzed intermolecular C(sp^3^)-H amidation. Angew. Chem. Int. Ed..

[59] Tang S., Peng P., Pi S.-F., Liang Y., Wang N.-X., Li J.H. (2008). Sequential intermolecular aminopalladation/*ortho*-arene C-H activation reactions of *N*-phenylpropiolamides with phthalimide. Org. Lett..

[60] Jordan-Hore J.A., Johansson C.C.C., Beck E.M., Gaunt M.J. (2008). Oxidative Pd(II)-catalyzed C–H bond amination to carbazole at ambient temperature. J. Am. Chem. Soc..

[61] Zakrzewski J., Smalley A.P., Kabeshov M.A., Gaunt M.J., Lapkin A.A. (2016). Continuous-Flow Synthesis and Derivatization of Aziridines through Palladium-Catalyzed C(sp^3^)–H Activation. Angew. Chem. Int. Ed..

[62] Yoshimura A., Zhdankin V.V. (2016). Advances in Synthetic Applications of Hypervalent Iodine Compounds. Chem. Rev..

[63] Canty A.J., Rodemann T., Skelton B.W., White A.H. (2006). Access to Alkynylpalladium(IV) and -Platinum(IV) Species, Including Triorgano(diphosphine)metal(IV) Complexes and the Structural Study of an Alkynyl(pincer)platinum(IV) Complex, Pt(O_2_CArF)I(C⋮CSiMe_3_)(NCN) (ArF = 4-CF_3_C_6_H_4_, NCN = [2,6-(dimethylaminomethyl)phenyl-*N*,C,*N*]-). Organometallics.

[64] Canty A.J., Patel J., Rodemann T., Ryan J.H., Skelton B.W., White A.H. (2004). Reactivity of Diaryliodine(III) Triflates toward Palladium(II) and Platinum(II): Reactions of C(sp^2^)–I Bonds to Form Arylmetal(IV) Complexes; Access to Dialkyl(aryl)metal(IV), 1,4-Benzenediyl-Bridged Platinum(IV), and Triphenylplatinum(IV) Species; and Structural Studies of Platinum(IV) Complexes. Organometallics.

[65] Bayler A., Canty A.J., Ryan J.H., Skelton B.W., White A.H. (2000). Arylation of palladium(II) and platinum(II) by diphenyliodonium triflate to form metal(IV) species, and a structural analysis of an isomer of PtIMe2Ph(bpy) (bpy = 2,2′-bipyridine). Inorg. Chem. Commun..

[66] Canty A.J., Rodemann T. (2003). Entry to alkynylplatinum(IV) chemistry using hypervalent iodine(III) reagents, and the synthesis of triphenyl{4,4′-bis(*tert*-butyl)-2,2′-bipyridine}iodoplatinum(IV). Inorg. Chem. Commun..

[67] Kalyani D., Deprez N.R., Desai L.V., Sanford M.S. (2005). Oxidative C–H activation/C–C bond forming reactions: Synthetic scope and mechanistic insights. J. Am. Chem. Soc..

[68] Wagner A.M., Sanford M.S. (2011). Palladium-catalyzed C-H arylation of 2,5-substituted pyrroles. Org. Lett..

[69] Deprez N.R., Sanford M.S. (2009). Synthetic and mechanistic studies of Pd-catalyzed C-H arylation with diaryliodonium salts: Evidence for a bimetallic high oxidation state Pd intermediate. J. Am. Chem. Soc..

[70] Canty A.J., Ariafard A., Sanford M.S., Yates B.F. (2013). Mechanism of Pd-Catalyzed Ar–Ar Bond Formation Involving Ligand-Directed C–H Arylation and Diaryliodonium Oxidants: Computational Studies of Orthopalladation at Binuclear Pd(II) Centers, Oxidation To Form Binuclear Palladium(III) Species, and Ar···Ar Reductive Coupling. Organometallics.

[71] Deprez N.R., Kalyani D., Krause A., Sanford M.S. (2006). Room temperature palladium-catalyzed 2-arylation of indoles. J. Am. Chem. Soc..

[72] Hickman A.J., Sanford M.S. (2011). Catalyst Control of Site Selectivity in the Pd II/IV-Catalyzed Direct Arylation of Naphthalene. ACS Catal..

[73] Tolnai G.L., Ganss S., Brand J.P., Waser J. (2012). C2-Selective Direct Alkynylation of Indoles. Org. Lett..

[74] Cho E.J., Senecal T.D., Kinzel T., Zhang Y., Watson D.A., Buchwald S.L. (2010). The Palladium-Catalyzed Trifluoromethylation of Aryl Chlorides. Science.

[75] Grushin V.V., Marshall W.J. (2006). Facile Ar–CF_3_ Bond Formation at Pd. Strikingly Different Outcomes of Reductive Elimination from [(Ph_3_P)_2_Pd(CF_3_)Ph] and [(Xantphos)Pd(CF_3_)Ph]. J. Am. Chem. Soc..

[76] Wang X., Truesdale L., Yu J.-Q. (2010). Pd(II)-Catalyzed ortho-Trifluoromethylation of Arenes Using TFA as a Promoter. J. Am. Chem. Soc..

[77] Ball N.D., Kampf J.W., Sanford M.S. (2010). Aryl–CF_3_ Bond-Forming Reductive Elimination from Palladium(IV). J. Am. Chem. Soc..

[78] Ball N.D., Gary J.B., Ye Y., Sanford M.S. (2011). Mechanistic and computational studies of oxidatively-induced aryl-CF_3_ bond-formation at Pd: Rational design of room temperature aryl trifluoromethylation. J. Am. Chem. Soc..

[79] Mu X., Chen S., Zhen X., Liu G. (2011). Palladium-Catalyzed Oxidative Trifluoromethylation of Indoles at Room Temperature. Chem. Eur. J..

[80] Powers D.C., Benitez D., Tkatchouk E., Goddard W.A., Ritter T. (2010). Bimetallic reductive elimination from dinuclear Pd(III) complexes. J. Am. Chem. Soc..

[81] Ye Y., Ball N.D., Kampf J.W., Sanford M.S. (2010). Oxidation of a cyclometalated Pd(II) dimer with “CF_3_+”: Formation and reactivity of a catalytically competent monomeric Pd(IV) aquo complex. J. Am. Chem. Soc..

[82] Powers D.C., Lee E., Ariafard A., Sanford M.S., Yates B.F., Canty A.J., Ritter T. (2012). Connecting binuclear Pd(III) and mononuclear Pd(IV) chemistry by Pd-Pd bond cleavage. J. Am. Chem. Soc..

[83] Cotton F.A., Gu J., Murillo C.A., Timmons D.J. (1998). The First Dinuclear Complex of Palladium(III). J. Am. Chem. Soc..

[84] Cotton F.A., Koshevoy I.O., Lahuerta P., Murillo C.A., Sanaú M., Ubeda M.A., Zhao Q. (2006). High Yield Syntheses of Stable, Singly Bonded Pd_2_^6+^ Compounds. J. Am. Chem. Soc..

[85] Powers D.C., Geibel M.A.L., Klein J.E.M.N., Ritter T. (2009). Bimetallic palladium catalysis: Direct observation of Pd(III)-Pd(III) intermediates. J. Am. Chem. Soc..

[86] Gol’dshleger N.F., Eskova V.V., Shilov A.E., Shteinman A.A. (1972). Alkane Reactions in Solutions of Chloride Complexes of Platinum. Zh. Fiz. Khim..

[87] Byers P.K., Canty A.J., Honeyman T. (1992). Conformational studies in Palladium(IV) and Platinum(IV) chemistry. Crystal structure of the 1,1-bis(pyrazol-1-yl)-ethane complex *fac*-PtIMe_3_[(pz)_2_CHMe-*N*,*N′*]. J. Orgmet. Chem..

[88] Canty A.J. (2009). Organopalladium and platinum chemistry in oxidising milieu as models for organic synthesis involving the higher oxidation states of palladium. Dalton Trans..

[89] Labinger J.A. (2016). Platinum-Catalyzed C–H Functionalization. Chem. Rev..

[90] Dick A.R., Kampf J.W., Sanford M.S. (2005). Platinum Model Studies for Palladium-Catalyzed Oxidative Functionalization of C–H Bonds. Organometallics.

[91] Whitfield S.R., Sanford M.S. (2008). Reactions of Platinum(II) Complexes with Chloride-Based Oxidants: Routes to Pt(III) and Pt(IV) Products. Organometallics.

[92] Mamtora J., Crosby S.H., Newman C.P., Clarkson G.J., Rourke J.P. (2008). Platinum(IV) Complexes: C–H Activation at Low Temperatures. Organometallics.

[93] Corbo R., Georgiou D.C., Wilson D.J.D., Dutton J.L. (2014). Reactions of [PhI(pyridine)_2_]^2+^ with model Pd and Pt II/IV redox couples. Inorg. Chem..

[94] Lanci M.P., Remy M.S., Lao D.B., Sanford M.S., Mayer J.M. (2011). Modulating Sterics in Trimethylplatinum(IV) Diimine Complexes To Achieve C–C Bond-Forming Reductive Elimination. Organometallics.

[95] Mutule I., Suna E., Olofsson K., Pelcman B. (2009). Catalytic direct acetoxylation of indoles. J. Org. Chem..

[96] Wagner A.M., Hickman A.J., Sanford M.S. (2013). Platinum-catalyzed C–H arylation of simple arenes. J. Am. Chem. Soc..

[97] Hashmi A.S.K. (2007). Gold-catalyzed organic reactions. Chem. Rev..

[98] Pflästerer D., Hashmi A.S.K. (2016). Gold catalysis in total synthesis—Recent achievements. Chem. Soc. Rev..

[99] Fürstner A., Davies P.W. (2007). Catalytic carbophilic activation: Catalysis by platinum and gold pi acids. Angew. Chem. Int. Ed. Engl..

[100] Wegner H.A., Auzias M. (2011). Gold for C-C coupling reactions: A Swiss-Army-knife catalyst?. Angew. Chem. Int. Ed..

[101] Komiya S., Kochi J.K. (1976). Electrophilic Cleavage of Organogold Complexes with Acids. The Mechanism of the Reductive Elimination of Dialkyl(aniono)gold. J. Am. Chem. Soc..

[102] Hopkinson M.N., Gee A.D., Gouverneur V. (2011). AuI/AuIII Catalysis: An Alternative Approach for C–C Oxidative Coupling. Chem. Eur. J..

[103] Wu C.-Y., Horibe T., Jacobsen C.B., Toste F.D. (2015). Stable Gold(III) catalysts by oxidative addition of a carbon-carbon bond. Nature.

[104] Wolf W.J., Winston M.S., Toste F.D. (2014). Exceptionally fast carbon-carbon bond reductive elimination from Gold(III). Nat. Chem..

[105] Winston M.S., Wolf W.J., Toste F.D. (2015). Halide-Dependent Mechanisms of Reductive Elimination from Gold(III). J. Am. Chem. Soc..

[106] Hashmi A.S.K., Blanco M.C., Fischer D., Bats J.W. (2006). Gold Catalysis: Evidence for the In-situ Reduction of Gold(III) During the Cyclization of Allenyl Carbinols. Eur. J. Org. Chem..

[107] Hashmi A.S.K., Ramamurthi T.D., Rominger F. (2009). Synthesis, structure and reactivity of organogold compounds of relevance to homogeneous gold catalysis. J. Organomet. Chem..

[108] Bennett M.A., Hockless D.C.R., Rae A.D., Welling L., Willis A.C. (2000). Carbon−Carbon Coupling in Dinuclear Cycloaurated Complexes Containing Bridging 2-(Diphenylphosphino)phenyl or 2-(Diethylphosphino)phenyl. Role of the Axial Ligand and the Fine Balance between Gold(II)–Gold(II) and Gold(I)–Gold(III). Organometallics.

[109] Zamora F., Amo-Ochoa P., Fischer B., Schimanski A., Lippert B. (1999). 5,5′-Diuracilyl Species from Uracil and. Angew. Chem. Int. Ed..

[110] Fuchita Y., Utsunomiya Y., Yasutake M. (2001). Synthesis and reactivity of Arylgold(III) complexes from aromatic hydrocarbons via C–H bond activation. J. Chem. Soc. Dalton Trans..

[111] Constable E.C., Sousa L.R. (1992). Metal-ion dependent reactivity of 2-(2′-thienyl)pyridine (Hthpy). J. Organomet. Chem..

[112] Huynh H.V., Guo S., Wu W. (2013). Detailed Structural, Spectroscopic, and Electrochemical Trends of Halido Mono- and Bis(NHC) Complexes of Au(I) and Au(III). Organometallics.

[113] Pažický M., Loos A., Ferreira M.J., Serra D., Vinokurov N., Rominger F., Jäkel C., Hashmi A.S.K., Limbach M. (2010). Synthesis, Reactivity, and Electrochemical Studies of Gold(I) and Gold(III) Complexes Supported by N-Heterocyclic Carbenes and Their Application in Catalysis. Organometallics.

[114] Gaillard S., Nun P., Slawin A.M.Z., Nolan S.P. (2010). Expeditious Synthesis of [Au(NHC)(L)]^+^ (NHC = *N*-Heterocyclic Carbene; L = Phosphine or NHC) Complexes. Organometallics.

[115] Hofer M., Nevado C. (2012). Unexpected Outcomes of the Oxidation of (Pentafluorophenyl)triphenylphosphanegold(I). Eur. J. Inorg. Chem..

[116] Ghidiu M.J., Pistner A.J., Yap G.P.A., Lutterman D.A., Rosenthal J. (2013). Thermal versus Photochemical Reductive Elimination of Aryl Chlorides from NHC–Gold Complexes. Organometallics.

[117] Orbisaglia S., Jacques B., Braunstein P., Hueber D., Pale P., Blanc A., de Frémont P. (2013). Synthesis, Characterization, and Catalytic Activity of Cationic NHC Gold(III) Pyridine Complexes. Organometallics.

[118] Corbo R., Pell T.P., Stringer B.D., Hogan C.F., Wilson D.J.D., Barnard P.J., Dutton J.L. (2014). Facile formation of homoleptic Au(III) trications via simultaneous oxidation and ligand delivery from [PhI(pyridine)_2_](2+). J. Am. Chem. Soc..

[119] Roşca D.-A., Smith D.A., Bochmann M. (2012). Cyclometallated Gold(III) hydroxides as versatile synthons for Au-N, Au-C complexes and luminescent compounds. Chem. Commun. (Camb.).

[120] Kar A., Mangu N., Kaiser H.M., Beller M., Tse M.K. (2008). A general gold-catalyzed direct oxidative coupling of non-activated arenes. Chem. Commun. (Camb.).

[121] Kar A., Mangu N., Kaiser H.M., Tse M.K. (2009). Gold-catalyzed direct oxidative coupling reactions of non-activated arenes. J. Organomet. Chem..

[122] Cambeiro X.C., Boorman T.C., Lu P., Larrosa I. (2013). Redox-controlled selectivity of C–H activation in the oxidative cross-coupling of arenes. Angew. Chem. Int. Ed..

[123] Ball L.T., Lloyd-Jones G.C., Russell C.A. (2012). Gold-catalyzed direct arylation. Science.

[124] Hofer M., Nevado C. (2013). Cross-coupling of arene–Gold(III) complexes. Tetrahedron.

[125] Haro T.D., Nevado C. (2010). Gold-Catalyzed Ethynylation of Arenes. J. Am. Chem. Soc..

[126] Brand J.P., Charpentier J., Waser J. (2009). Direct alkynylation of indole and pyrrole heterocycles. Angew. Chem. Int. Ed..

[127] Brand J.P., Li Y., Waser J. (2013). Gold-Catalyzed Alkynylation: Acetylene-Transfer instead of Functionalization. Isr. J. Chem..

[128] Brand J.P., Waser J. (2010). Direct alkynylation of thiophenes: Cooperative activation of TIPS-EBX with gold and Brønsted acids. Angew. Chem. Int. Ed..

[129] Brand J.P., Waser J. (2012). Para-Selective Gold-Catalyzed Direct Alkynylation of Anilines. Org. Lett..

[130] Li Y., Brand J.P., Waser J. (2013). Gold-catalyzed regioselective synthesis of 2- and 3-alkynyl furans. Angew. Chem. Int. Ed. Engl..

[131] Iglesias A., Muñiz K. (2009). Oxidative Interception of the Hydroamination Pathway: A Gold-Catalyzed Diamination of Alkenes. Chem. Eur. J..

[132] Zhang D.-H., Dai L.-Z., Shi M. (2010). C(sp^3^)–C(sp^3^) Bond Breaking in Methylenecyclopropanes Involving a AuI/AuIII Catalytic Cycle. Eur. J. Org. Chem..

[133] Ananikov V.P. (2015). Nickel: The “Spirited Horse” of Transition Metal Catalysis. ACS Catal..

[134] Klein H.-F., Bickelhaupt A., Jung T., Cordier G. (1994). Syntheses and Properties of the First Octahedral Diorganonickel(IV) Compounds. Organometallics.

[135] Shimada S., Rao M., Tanaka M. (1999). Reaction of 1,2-Disilylbenzene with Bis[1,2-bis(dimethylphosphino)ethane]nickel(0). Isolation and Characterization of the First Silylnickel(IV) Complex—Organometallics (ACS Publications). Organometallics.

[136] Dimitrov V., Linden A. (2003). A pseudotetrahedral, high-oxidation-state organonickel compound: Synthesis and structure of bromotris(1-norbornyl)nickel(IV). Angew. Chem. Int. Ed. Engl..

[137] Carnes M., Buccella D., Chen J.Y.-C., Ramirez A.P., Turro N.J., Nuckolls C., Steigerwald M. (2009). A stable tetraalkyl complex of nickel(IV). Angew. Chem. Int. Ed..

[138] Higgs A.T., Zinn P.J., Simmons S.J., Sanford M.S. (2009). Oxidatively Induced Carbon–Halogen Bond-Forming Reactions at Nickel. Organometallics.

[139] Higgs A.T., Zinn P.J., Sanford M.S. (2010). Synthesis and Reactivity of NiII(Phpy)_2_ (Phpy = 2-Phenylpyridine). Organometallics.

[140] Camasso N.M., Sanford M.S. (2015). Design, synthesis, and carbon-heteroatom coupling reactions of organometallic Nickel(IV) complexes. Science.

[141] Bour J.R., Camasso N.M., Sanford M.S. (2015). Oxidation of Ni(II) to Ni(IV) with Aryl Electrophiles Enables Ni-Mediated Aryl-CF_3_ Coupling. J. Am. Chem. Soc..

[142] Meucci E.A., Camasso N.M., Sanford M.S. (2017). An Organometalllic Ni(IV) Complex That Participates in Competing Transmetalation and C(sp^2^)–O Bond-Forming Reductive Elimination Reactions. Organometallics.

[143] Martinez G.E., Ocampo C., Park Y.J., Fout A.R. (2016). Accessing Pincer Bis(carbene) Ni(IV) Complexes from Ni(II) via Halogen and Halogen Surrogates. J. Am. Chem. Soc..

[144] Hwang S.J., Powers D.C., Maher A.G., Anderson B.L., Hadt R.G., Zheng S.-L., Chen Y.-S., Nocera D.G. (2015). Trap-Free Halogen Photoelimination from Mononuclear Ni(III) Complexes. J. Am. Chem. Soc..

[145] Yokota A., Aihara Y., Chatani N. (2014). Nickel(II)-Catalyzed Direct Arylation of C–H Bonds in Aromatic Amides Containing an 8-Aminoquinoline Moiety as a Directing Group. J. Org Chem..

[146] Lee E., Hooker J.M., Ritter T. (2012). Nickel-Mediated Oxidative Fluorination for PET with Aqueous [18F] Fluoride. J. Am. Chem. Soc..

[147] Willert-Porada M.A., Burton D.J., Baenziger N.C. (1989). Synthesis and X-ray structure of bis(trifluoromethyl)(*N*, *N*-diethyldithiocarbamato)-copper; a remarkably stable perfluoroalkylcopper(III) complex. J. Chem. Soc. Chem. Commun..

[148] Furuta H., Maeda H., Osuka A. (2000). Doubly N-Confused Porphyrin: A New Complexing Agent Capable of Stabilizing Higher Oxidation States. J. Am. Chem. Soc..

[149] Santo R., Miyamoto R., Tanaka R., Nishioka T., Sato K., Toyota K., Obata M., Yano S., Kinoshita I., Ichimura A. (2006). Diamagnetic-paramagnetic conversion of tris(2-pyridylthio)methylcopper(III) through a structural change from trigonal bipyramidal to octahedral. Angew. Chem. Int. Ed. Engl..

[150] Phipps R.J., Gaunt M.J. (2009). A meta-selective copper-catalyzed C–H bond arylation. Science.

[151] Phipps R.J., Grimster N.P., Gaunt M.J. (2008). Cu(II)-catalyzed direct and site-selective arylation of indoles under mild conditions. J. Am. Chem. Soc..

[152] Parsons A.T., Buchwald S.L. (2011). Copper-Catalyzed Trifluoromethylation of Unactivated Olefins. Angew. Chem. Int. Ed..

[153] Filippou A.C., Winter J.G., Kociok-Köhn G., Troll C. (1999). Electron-Rich Trichlorogermyl Complexes of Molybdenum and Tungsten Bearing a Cyclopentadienyl Ligand: Synthesis, Crystal Structures, and Cyclic Voltammetric Studies. Organometallics.

[154] Hayton T.W., Legzdins P., Patrick B.O. (2002). Differing reactivities of (trimpsi)M(CO)(2)(NO) complexes [M = V, Nb, Ta; trimpsi = (*t*)BuSi(CH(_2_)PMe(_2_))(_3_)] with halogens and halogen sources. Inorg. Chem..

[155] Dröse P., Crozier A.R., Lashkari S., Gottfriedsen J., Blaurock S., Hrib C.G., Maichle-Mössmer C., Schädle C., Anwander R., Edelmann F.T. (2010). Facile access to tetravalent cerium compounds: One-electron oxidation using iodine(III) reagents. J. Am. Chem. Soc..

[156] Crispini A., Ghedini M., Neve F. (1993). Synthesis and structure of the dimer Ir_2_Cl_2_I_2_(CO)_2_(μ-dppm )_2_]. Inorg. Chim. Acta.

[157] Teets T.S., Nocera D.G. (2012). Oxygen reduction reactions of monometallic rhodium hydride complexes. Inorg. Chem..

[158] Young K.J.H., Mironov O.A., Periana R.A. (2007). Stoichiometric Oxy Functionalization and CH Activation Studies of Cyclometalated Iridium(III) 6-Phenyl-2,2′-Bipyridine Hydrocarbyl Complexes. Organometallics.

[159] Li Z. (2004). Oxidation of hydrocarbons with iodobenzene diacetate catalyzed by manganese(III) porphyrins in a room temperature ionic liquid. J. Mol. Catal. A Chem..

[160] Karimipour G.R., Shadegan H.A., Ahmadpour R. (2007). Clean and highly selective oxidation of alcohols by the PhI(OAc)_2_/Mn(TPP)CN/Im catalytic system. J. Chem. Res..

[161] In J.-H., Park S.-E., Song R., Nam W. (2003). Iodobenzene diacetate as an efficient terminal oxidant in Iron(III) porphyrin complex-catalyzed oxygenation reactions. Inorg. Chim. Acta.

[162] Fumagalli G., Boyd S., Greaney M.F. (2013). Oxyarylation and aminoarylation of styrenes using photoredox catalysis. Org. Lett..

[163] Lu M.-Z., Loh T.-P. (2014). Iron-catalyzed cascade carbochloromethylation of activated alkenes: Highly efficient access to chloro-containing oxindoles. Org. Lett..

